# Wwp2 maintains cartilage homeostasis through regulation of Adamts5

**DOI:** 10.1038/s41467-019-10177-1

**Published:** 2019-06-03

**Authors:** Sho Mokuda, Ryo Nakamichi, Tokio Matsuzaki, Yoshiaki Ito, Tempei Sato, Kohei Miyata, Masafumi Inui, Merissa Olmer, Eiji Sugiyama, Martin Lotz, Hiroshi Asahara

**Affiliations:** 1 Department of Molecular Medicine, The Scripps Research Institute, 10550 North Torrey Pines Rd., La Jolla, CA 92037 USA; 2 Department of Clinical Immunology and Rheumatology, Hiroshima University Hospital, 1-2-3 Kasumi, Minami-ku, Hiroshima, 734-8551 Japan; 3 Department of Systems BioMedicine, Graduate School of Medical and Dental Sciences, Tokyo Medical and Dental University (TMDU), 1-5-45 Yushima, Bunkyo-ku, Tokyo, 113-8510 Japan; 4 Research Core, Research Facility Cluster, Institute of Research, Tokyo Medical and Dental University (TMDU), 1-5-45 Yushima, Bunkyo-ku, Tokyo, 113-8510 Japan; 5 Laboratory of Animal Regeneration Systemology, Department of Life Sciences, School of Agriculture, Meiji University, 1-1-1 Higashimita, Tama-ku, Kawasaki, Kanagawa 214-8571 Japan; 6 Meiji University International Institute for Bio-Resource Research, 1-1-1 Higashimita, Tama-ku, Kawasaki, Kanagawa 214-8571 Japan

**Keywords:** CRISPR-Cas9 genome editing, miRNAs, Ubiquitylation, Osteoarthritis

## Abstract

The *WW domain-containing protein 2* (*Wwp2*) gene, the host gene of miR-140, codes for the Wwp2 protein, which is an HECT-type E3 ubiquitin ligases abundantly expressed in articular cartilage. However, its function remains unclear. Here, we show that mice lacking Wwp2 and mice in which the Wwp2 E3 enzyme is inactivated (Wwp2-C838A) exhibit aggravated spontaneous and surgically induced osteoarthritis (OA). Consistent with this phenotype, WWP2 expression level is downregulated in human OA cartilage. We also identify Runx2 as a Wwp2 substrate and Adamts5 as a target gene, as similar as miR-140. Analysis of Wwp2-C838A mice shows that loss of Wwp2 E3 ligase activity results in upregulation of Runx2-Adamts5 signaling in articular cartilage. Furthermore, in vitro transcribed Wwp2 mRNA injection into mouse joints reduces the severity of experimental OA. We propose that Wwp2 has a role in protecting cartilage from OA by suppressing Runx2-induced Adamts5 via Runx2 poly-ubiquitination and degradation.

## Abstract (web summary)

Wwp2 is an HECT-type E3 ubiquitin ligase abundantly expressed in articular cartilage. Here, the authors show that in mice, loss of Wwp2 leads to upregulated Runx2-Adamts5 signaling in articular cartilage and development of osteoarthritis, and that disease severity is reduced by injection of Wwp2 mRNA

## Introduction

Osteoarthritis (OA) is the most common joint disease and leads to chronic disability in elderly patients. Destruction of articular hyaline cartilage in the synovial joints is a key event in OA initiation and progression^1–4^. Adult articular cartilage consists of chondrocytes and extracellular matrix (ECM), such as collagens and proteoglycans including Aggrecan. Under physiological conditions, chondrocytes maintain the equilibrium of ECM and cartilage homeostasis by producing anabolic molecules^5^. During aging and joint diseases, catabolic reactions against ECM in cartilage dominate, leading to loss of ECM proteins and contributing to cartilage destruction^5^.

ADAMTS (A disintegrin and metalloproteinase with thrombospondin motifs) is a family of 19 secreted extracellular protease enzymes, which can cleave procollagens, neurocan, aggrecan, brevican and versican^6^. ADAMTS4 and ADAMTS5 (Aggrecanase-1 and Aggrecanase-2, respectively) consist of a catalytic metalloprotease domain and a series of other ancillary domains, which participate in regulating their activity and substrate specificity^7^. These proteins are involved in the excessive matrix degradation that characterizes cartilage damage in OA^8,9^. Expression levels of ADAMTS4 in human OA cartilage are higher than in normal cartilage^10^. It has been reported that Adamts5 is an important enzyme in OA pathogenesis because gene deletion prevented cartilage degradation in murine OA models^11,12^.

Wwp2, WW domain-containing protein 2, is a member of the C2-WW-HECT family (NEDD4 family) of the E3 ubiquitin ligases (E3)^13^, which act as acceptors of ubiquitin from E2 enzymes and then transfer ubiquitin to a specific lysine residue on the substrate^14^. miR-140 is located in the *Wwp2* locus as an intronic microRNA^15^. Both Wwp2 and miR-140 are abundantly and specifically expressed in cartilage^16,17^, suggesting a potential function in this tissue. Our group and others previously reported that miR-140 plays a critical role both in craniofacial development and cartilage homeostasis^18–20^, whereas Wwp2 knockout (KO) (*Wwp2*^*1ins/1ins*^) mice did not show craniofacial deformities^21^, implicating low relevance of Wwp2 during development. Wwp2 function and its substrate in adult cartilage homeostasis and OA remain unclear. Here we report that E3 ligase activity of Wwp2 maintains cartilage homeostasis through regulation of Runx2-Adamts5 signaling. We propose that Wwp2 replacement is a novel therapeutic approach for cartilage destruction, as similar as miR-140.

## Results

### Loss of Wwp2 exacerbates articular cartilage destruction

**Fig. 1:**
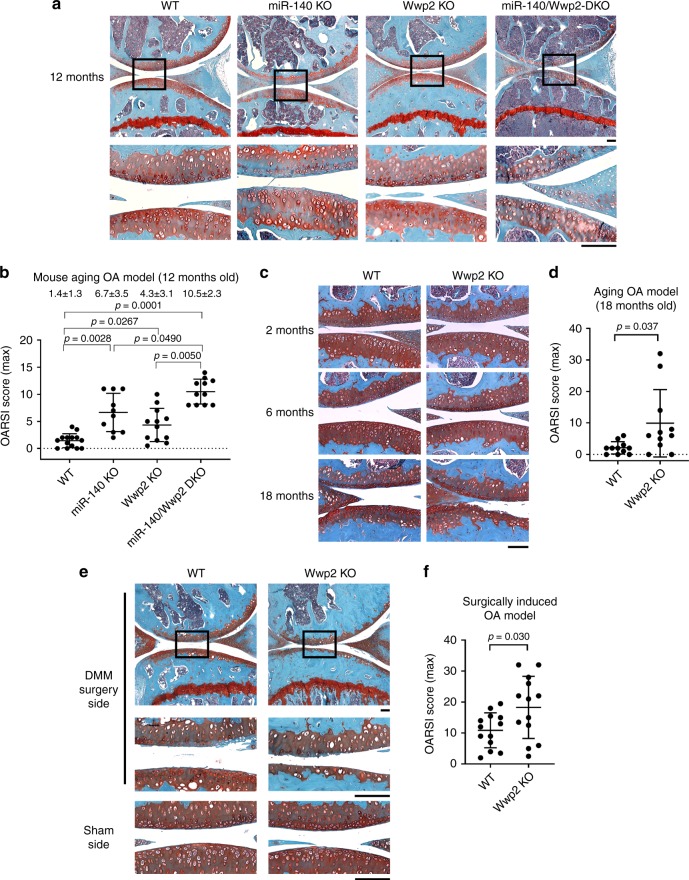
Loss of Wwp2 exacerbates articular cartilage destruction. **a**, **b**. Results of aging (12-month-old) OA murine model compared among wild type (WT), miR-140 KO (*miR-140*^*14del/14del*^), Wwp2 KO (*Wwp2*^1ins/1ins^) and miR-140/Wwp2-DKO (*Wwp2*^*1del/1del*^; *miR-140*^*132del/132del*^) mice. **a** Representative images of Safranin-O staining. **b** The OARSI scores (*n* = 10-14, Dunn test compared with WT and DKO, respectively). **c** Representative images of Safranin-O staining of WT and Wwp2 KO mice (2, 6, 18-month-old). **d** The OARSI scores of WT and Wwp2 KO mice in 18-month-old (*n* = 11, Welch’s *t* test). **e**, **f** Results of surgically (DMM) induced OA murine model compared to WT and Wwp2 KO mice. **e** Representative images of Safranin-O staining. **f** The maximum OARSI scores of WT and Wwp2 KO mice (*n* = 13, Student’s *t* test). Black scale bar = 1 mm. Source data are provided as a Source Data file. Data are presented as the mean ± SD

The *Wwp2* locus can generate both miR-140, an intronic microRNA, and Wwp2 protein (Supplementary Fig. 1A). Our group has previously reported that deletion of miR-140 in mice caused early onset of the OA phenotype^18^. To examine whether Wwp2 is related to cartilage homeostasis and OA pathogenesis, we examined aging-related changes (12 months old) in knee articular cartilages of miR-140 and Wwp2 individual and double KO (DKO) mice which were generated using the CRISPR/Cas9 system:^21^ miR-140 KO (*miR-140*^*14del/14del*^), Wwp2 KO (*Wwp2*^1ins/1ins^) and miR-140/Wwp2-DKO (*Wwp2*^*1del/1del*^; *miR-140*^*132del/132del*^) (Supplementary Fig. 1A). DKO mice exhibited more severe OA changes than Wwp2 KO or miR-140 KO mice, which showed more severe OA changes compared to in WT mice (Fig. 1a, b) (Supplementary Fig. 1B, C, D, E). The OA changes in Wwp2 KO mice were observed at 12 months, although these changes are milder rather than in miR-140/Wwp2-DKO mice. To further analyze the phenotype of articular cartilage in Wwp2 KO mouse, we also investigated aging-related OA at 18 months and surgically induced OA model. In the 18-month-aging model and the surgical destabilization of the medial meniscus (DMM) model of OA, a more severe OA phenotype in Wwp2 KO mice was observed compared to the WT mice (Fig. 1c–f) (Supplementary Fig. 2A). Wwp2 protein expression levels of these Wwp2 KO mice decreased, while miR-140 expression levels were unchanged (Supplementary Fig. 1B, D). These mice showed no obvious developmental abnormalities, such as craniofacial malformation, short body length, change in growth plate dimensions, or irregularity in articular bone alignment (Supplementary Fig. 3A, C, F, H). These data suggest that Wwp2 has a protective function against cartilage destruction.

### Loss of Wwp2 during aging, injury or inflammation

**Fig. 2:**
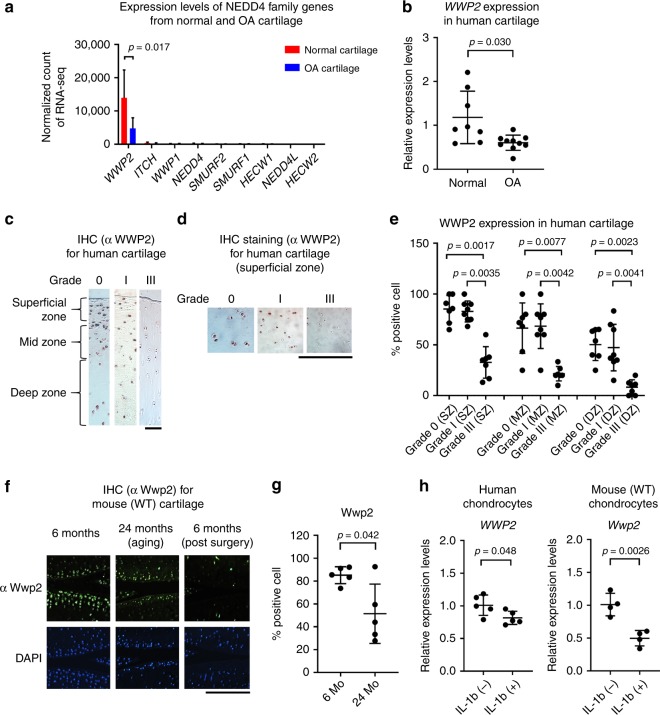
Loss of Wwp2 during aging, injury or inflammation. **a** Human samples were classified using a modified Outer bridge scale (grade 0-IV). RNA-seq analyses of articular cartilage to compare normal cartilage (grade ≤ I) and OA cartilage (grade ≥ III). After the data were normalized, the counts of the NEDD4 family were sorted. (*n* = 8-10, Welch’s *t* test). The ages of subjects are following: normal cartilage, 37.8 ± 13.0 years-old; OA cartilage, 62.7 ± 7.5 years-old, respectively. **b** RT-qPCR analyses of articular cartilage to compare normal and OA cartilage (*n* = 8-10, Welch’s *t* test, normalized with *GAPDH*). The ages of subjects are follows: normal cartilage, 50.5 ± 11.5 years-old; OA cartilage, 62.9 ± 8.2 years-old. **c**–**e** IHC staining of WWP2 in human articular cartilage. **c**, **d** Classification of zones and representative images. Wwp2 was stained with AEC (red). **e** WWP2 positive cell rate (*n* = 7–8, Dunn test compared with Grade III). The ages of subjects are as follows: grade 0, 18.7 ± 1.7 years-old; Grade I, 44.8 ± 18.8 years-old; Grade III, 70.9 ± 20.0 years-old, respectively. **f** Representative images of IHC staining of Wwp2 in wild-type (WT) mouse articular cartilage, detected as green (Wwp2) and blue (DAPI, nuclei), to compare between 6-month-old and 24-month-old (aging OA). Right images are IHC staining for surgically (DMM) induced OA specimens. **g** Wwp2 positive cell rate (*n* = 5, Welch’s *t* test). **h** RT-qPCR for human and mouse articular chondrocytes stimulated with IL-1β (10 ng/ml) (*n* = 5 and 4, Student’s *t* test, normalized with *GAPDH* and *Gapdh*, respectively). Black scale bar = 1 mm. Source data are provided as a Source Data file. Data are presented as the mean ± SD

Next, WWP2 and Wwp2 expressions were examined in human and mouse OA and normal articular cartilages. In OA human articular cartilage, WWP2 expression levels were lower than in normal tissue as examined by RNA-seq or reverse transcription (RT)-quantitative polymerase chain reaction (qPCR) (Fig. 2a, b) (Supplementary Table 2), and immunohistochemistry (IHC) (Fig. 2c–e) (Supplementary Fig. 4A) (Supplementary Table 2). In mouse articular cartilage, Wwp2 expression was lower in aging or surgically induced OA (Fig 2f, g). *WWP2* and *Wwp2* expression levels in human and mouse primary cultured chondrocytes, respectively, were suppressed by interleukin (IL)-1β stimulation, a mediator in OA pathogenesis^22^ (Fig. 2h).

### Wwp2 regulates Adamts5 expression in articular cartilage

**Fig. 3:**
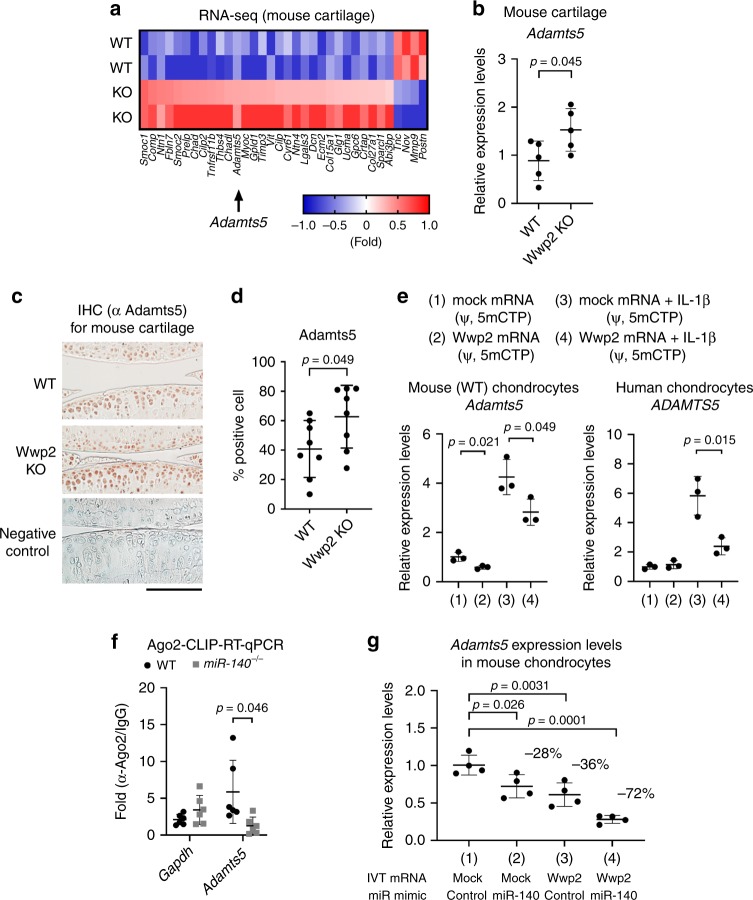
Wwp2 regulates Adamts5 expression in articular cartilage. **a** RNA-seq analyses of 2-month-old articular cartilage comparing WT and Wwp2 KO mice. Each samples were isolated from three individual mice. After normalization, Genes with count values ≥ 2000 and a change ≥ 1.5-log2fold were extracted and used for analysis. A heat map showing proteinaceous extracellular matrix related genes, classified by DAVID analysis. **b** RT-qPCR for 2-month-old mouse articular cartilage (*n* = 5, Student’s *t* test, normalized with *Gapdh*). **c**, **d** IHC staining of Adamts5 for mouse articular cartilage (6-month-old). **c** Representative images. Adamts5 was stained with AEC (red). Negative control was stained using rabbit control IgG. **d** Adamts5 positive cell rate (*n* = 8, Student’s *t* test). **e** Wwp2 overexpression experiments using in vitro transcribed (IVT) mRNA (ψ, 5mCTP) with or without IL-1β (10 ng/mL, 17 h) for mouse and human chondrocytes, whose *Adamts5* and *ADAMTS5* expression levels were analyzed by RT-qPCR (*n* = 3, Student’s *t* test, normalized with *Gapdh* and *GAPDH*, respectively). **f** Ago2-crosslinking immunoprecipitation (CLIP) analyses of primary chondrocytes to detect Ago2-binding mRNAs, compared between WT and miR-140^-/-^ mice (*n* = 3, Welch’s *t* test). The primers were designed for the 3′ untranslated region of *Adamts5* gene and a coding region of *Gapdh*. **g** Double overexpression experiments using IVT Wwp2 mRNA (ψ, 5mCTP) and miR-140 mimic in mouse chondrocytes. RT-qPCR for *Adamts5* expression levels (*n* = 4, Dunnett test compared with WT and DKO, respectively, normalized with *Gapdh*). Black scale bar = 1 mm. Source data are provided as a Source Data file. Data are presented as the mean ± SD

To further investigate the function of Wwp2 in articular cartilage, we performed RNA-seq analysis using mouse articular cartilages from WT and Wwp2 KO mice (Fig. 3a). In gene ontology analysis, the expression of 33 genes classified as ECM-related factors were dysregulated in Wwp2 KO mice. Among the upregulated genes, we focused on Adamts5, since there is genetic evidence linking Adamts5 and articular cartilage destruction^11,12^. *Adamts5* were higher levels in Wwp2 KO mice than in WT mice (Fig. 3a, b) (Supplementary Fig. 4B, C, D). Elevated Adamts5 protein expression was confirmed by IHC in the articular cartilage of Wwp2 KO mice compared to in WT mice (Fig. 3c, d). To further investigate the relationship between Adamts5 and Wwp2, we performed overexpression experiments using in vitro transcribed (IVT) mRNA, which contained modified nucleic acids (pseudouridine-5′-triphosphate (ψ) and 5-methylcytidine-5′-triphosphate (5mCTP)) to reduce the inflammatory response against single-stranded RNA^23,24^ (Supplementary Fig. 4E). When we transfected IVT Wwp2 mRNA (ψ, 5mCTP) into mouse chondrocytes, *Adamts5* expression levels were downregulated (Fig. 3e) (Supplementary Fig. 4F). In human chondrocytes, *ADAMTS5* expression induced by IL-1β stimulation was also downregulated by Wwp2 overexpression (Fig. 3e) (Supplementary Fig. 4G). Therefore, these data indicated that Wwp2 regulates Adamts5 expression in articular cartilage.

The Wwp2 gene encodes both miR-140 and Wwp2 (Supplementary Fig. 1A). As previously reported, one of targets of miR-140 was Adamts5^18^, similar to Wwp2. Thus, we examined functional interaction between Wwp2 and miR-140. To test miR-140 binding activity, Ago2-CLIP-qPCR analysis showed that the target mRNA levels of *Adamts5* in Ago2-binding mRNAs were more concentrated in WT chondrocytes than in miR-140^-/-^ chondrocytes (Fig. 3f)^18^, suggesting that miR-140 directly binds to the Adamts5 mRNAs. Treatment with both IVT Wwp2 mRNA (ψ, 5mCTP) and miRNA-140 mimic showed that *Adamts5* expression levels were lowest when both Wwp2 and miR-140 were administered simultaneously (Fig. 3g), implying the functional cooperativity between Wwp2 and miR-140 in chondrocytes.

### Wwp2 regulates Adamts5 through poly-ubiquitination of Runx2

**Fig. 4:**
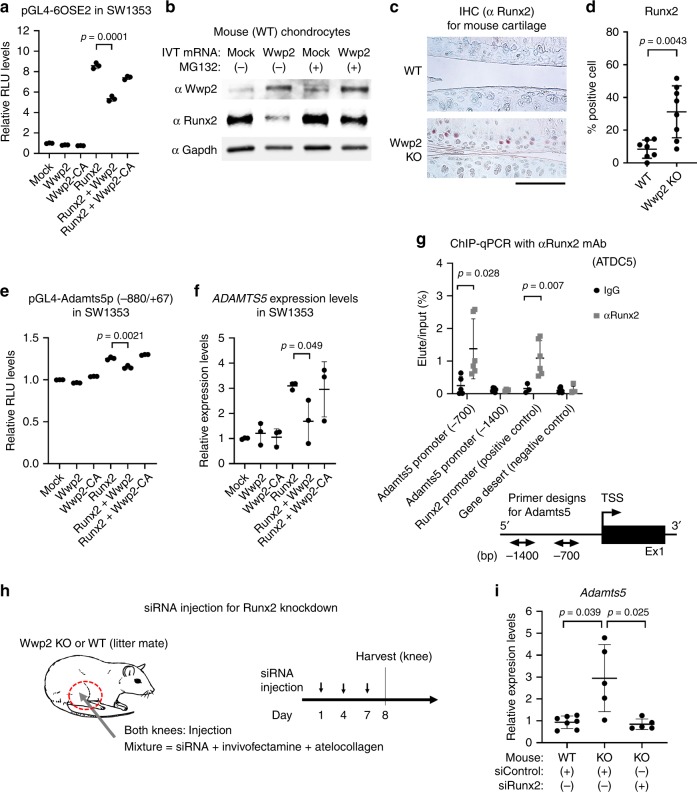
E3 ligase activity of Wwp2 regulates Adamts5 through degradation of Runx2. **a** Runx2 reporter luciferase assay (pGL4-6OSE2) using SW1353 cells transfected with plasmids (Runx2, Wwp2, Wwp2-C838A (Wwp2-CA)) (*n* = 3, Student’s *t* test). **b** Mouse chondrocytes were transfected with IVT Wwp2 mRNA (ψ, 5mCTP) and treated with protease inhibitor MG132 (2 μM, 60 minutes). This experiment was performed two or more times. Representative images are shown. **c**, **d** IHC of Runx2 for mouse articular cartilage (6-month-old). **c** Representative images. Runx2 was stained with Vector Red (red). **d** Runx2 positive cell rate (*n* = 7–8, Welch’s *t* test). **e** Adamts5 promoter luciferase assay (pGL4-Adamts5p (-880/ + 67)) in SW1353 cells transfected with plasmids (Runx2, Wwp2, Wwp2-CA) (*n* = 3, Student’s *t* test). **f** RT-qPCR for *ADAMTS5* expression in SW1353 cells transfected with plasmids (Runx2, Wwp2, Wwp2-CA) (*n* = 3, Student’s *t* test, normalized with *GAPDH*). **g** ChIP-qPCR using anti-Runx2 antibody and ATDC5 cell line. The primer pairs enhanced Adamts5 promoter region (-700 or -1400 relative to the TSS), Runx2 promoter region (positive control) and gene desert (negative control) (*n* = 6, Welch’s *t* test). **h**, **i** The in vivo knockdown experiment using siRunx2. Invivofectamine, atelocollagen and siRNA (siRunx2 and siControl) were injected into mouse knees. **h** Schedule of in vivo transfection. **i** RT-qPCR for *Adamts5* expression levels in mouse cartilage (*n* = 5-7, Dunn test compared with Wwp2 KO mouse treated with siControl, normalized with *Gapdh*). Black scale bar = 1 mm. Source data are provided as a Source Data file. Data are presented as the mean ± SD

The molecular mechanism of how Wwp2 reduces Adamts5 has not been determined previously. Adamts5 was excluded as a Wwp2 substrate because Adamts5 does not have a PY-motif^13,25^. In this regard, we searched the upstream signals and trans-acting factors of Adamts5 for Wwp2 substrates. This search identified Runt-related transcription factor 2 (Runx2). Adamts5 expression in chondrocytes and OA pathogenesis has been reported to be enhanced by nuclear factor-kappa B (NF-κB) or Runx2^26^. It has also been reported that RUNX2 can up-regulate ADAMTS5 using real-time PCR, luciferase assay, and chromatin immunoprecipitation (ChIP) analyses^27,28^. We performed luciferase assays using Runx2 reporter or NF-κB reporter and found that Wwp2 overexpression repressed Runx2 reporter activity, but not NF-κB reporter activity (Fig. 4a) (Supplementary Fig. 5A). Overexpression of Wwp2 decreased Runx2 protein levels, while MG132, a proteasome inhibitor, blocked Runx2 degradation (Fig. 4b). And, elevated Runx2 protein levels were detected in the articular cartilage of Wwp2 KO mice compared to in WT mice (Fig. 4c, d). In addition, small interfering RNA (siRNA) against Wwp2 (siWwp2) upregulated Runx2 transcriptional activity (Supplementary Fig. 5B, C). These data suggest that Wwp2 might induce Runx2 protein degradation through the ubiquitin-proteasome system.

In Wwp2-C838A (Wwp2-CA), the crucial cysteine-838 residue was mutated to alanine to inactivate its E3 ligase activity^14^. In a cycloheximide (CHX) blockade experiment, Wwp2 promoted Runx2 degradation compared to the Wwp2-CA control (Supplementary Fig. 5D). A luciferase assay using mouse Adamts5 promoter region (from -880 to + 67 base pairs (bp) relative to the transcription start site (TSS)) and RT-qPCR for *Adamts5* expression showed that Wwp2 regulated Runx2-induced *Adamts5* expression while Wwp2-CA did not (Fig. 4e, f). The luciferase assay using siWwp2 revealed the upregulation of Adamts5 expression (Supplementary Fig. 5C). To confirm the binding activity of Runx2 to the responsive DNA sequences, ChIP-qPCR was performed using an anti-Runx2 antibody in the ATDC5 chondrogenic cell line. The results indicated that Runx2 bound to the Adamts5 promoter region (approximately −700 bp relative to the TSS) (Fig. 4g). Furthermore, the observation that siRNA against Runx2 (siRunx2) downregulated Adamts5 expression is supporting this relationship (Supplementary Fig. 5C).

**Fig. 5:**
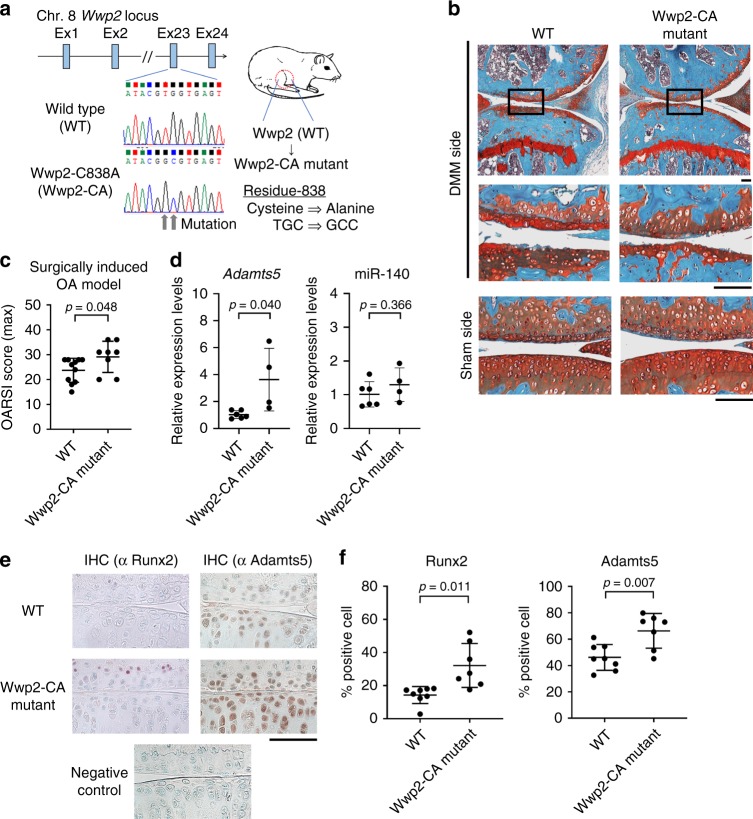
Wwp2-C838A (E3 ligase inactivated) mouse promotes articular cartilage destruction. **a** Schema of Wwp2-CA (*Wwp2*^*C838A/C838A*^) mutant mouse. **b**, **c** Results of surgically (DMM) induced OA murine model compared to WT and Wwp2-CA mutant mice. **b** Representative images of Safranin-O staining. **c** The maximum OARSI scores of WT and Wwp2-CA mutant mice (*n* = 8-11, Student’s *t* test). **d** RT-qPCR for *Adamts5* and miR-140 expression in mouse articular cartilage comparing wild type (WT) and Wwp2-CA mice (2-month-old). (*n* = 4-6, Welch’s *t* test, normalized with *Gapdh* and U6snRNA, respectively). **e**, **f** IHC of Runx2 and Adamts5 for mouse articular cartilage (6-month-old). **e** Representative images. Negative control was stained using rabbit control IgG. **f** Runx2 and Adamts5 positive cell rates (*n* = 7–8, Welch’s *t* test). Black scale bar = 1 mm. Source data are provided as a Source Data file. Data are presented as the mean ± SD

To test these interactions (Wwp2-Runx2-Adamts5 axis) in vivo, we performed two experiments. First, we treated Wwp2 KO mice with siRunx2 by injection into the knee joints (Fig. 4h). Results showed that siRunx2 reduced Adamts5 expression in articular cartilage of the Wwp2 KO mice (Fig. 4i). Second, we generated Wwp2-CA (*Wwp2*^*C838A/C838A*^) mutant mouse using the CRISPR/Cas9 system (Fig. 5a) (Supplementary Fig. 6A, B)^29^. In agreement with our recent findings^21^, we did not detect developmental abnormalities in this mouse line (Supplementary Fig. 3B, D, G, I). These Wwp2 enzyme inactivated mice developed more severe surgically induced OA compared to WT mice (Fig. 5b, c) (Supplementary Fig. 2B). *Adamts5* expression levels in the knee cartilage were also higher than those in WT mice, while their miR-140 expression levels remained unchanged (Fig. 5d). Runx2 and Adamts5 expression levels in articular cartilages from Wwp2-CA mice were elevated compared to WT mice, as detected by IHC (Fig. 5e, f). These findings further support the notion that the E3 ligase activity of Wwp2 is crucial for repressing the Runx2-Adamts5 signaling pathway.

**Fig. 6:**
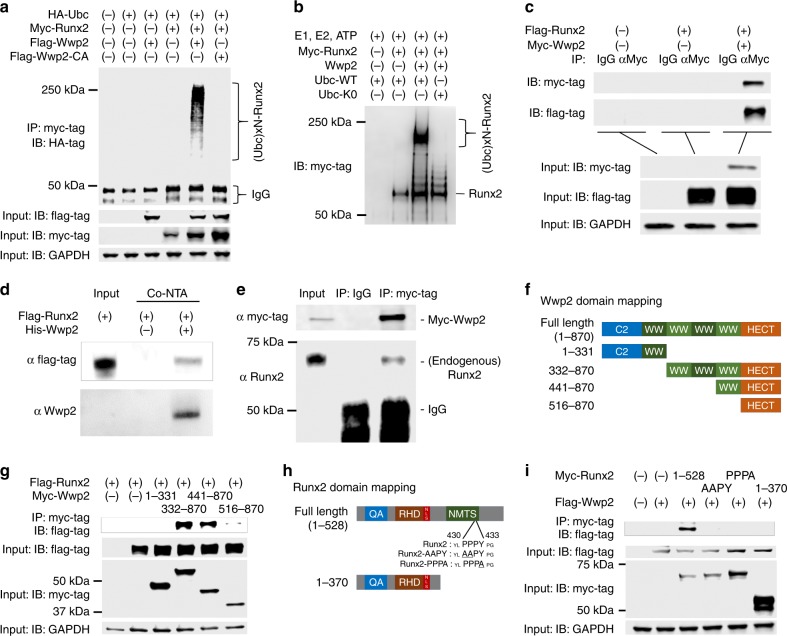
Wwp2 induces poly-ubiquitination of Runx2. **a** An immunoprecipitation (IP) experiment to detect ubiquitinated Runx2. HEK293T cells were transfected with plasmids (HA-ubiquitin (Ubc), myc (or flag)-Runx2, flag (or myc)-Wwp2, flag-Wwp2-CA) and treated with MG132 (10 μM, 7 h). The cell lysates were treated by anti-myc-tag antibody and protein A. **b** In vitro ubiquitination assay to demonstrate Wwp2-induced poly-ubiquitination. Wwp2 and Runx2 proteins were purified from transfected HEK293T cells. **c** A Co-IP experiment to detect binding activity between Runx2 and Wwp2. HEK293T cells were transfected with plasmids and treated with MG132. The cell lysates were treated by anti-myc-tag antibody and protein A. **d** In vitro pull-down assay using His-tag. Proteins were purified from transfected HEK293T cells. **e** Myc-Wwp2 transduced ATDC5 chondrogenic cell line was used. Co-IP using anti-myc-tag antibody indicated Wwp2 bound with endogenous Runx2. **f** Schema of Wwp2 fragments for domain mapping. **g** Co-IP to confirm the binding domain of Wwp2 for Runx2. **h** Schema of Runx2 fragments and mutations for domain mapping. For the PY motif, mutations were introduced in PPPY sequence, such as AAPY or PPPA. **i** Co-IP to detect the binding sequence of Runx2 for Wwp2. These experiments were performed two or more times. Representative images are shown. Source data are provided as a Source Data file. C2, C2 domain; WW, WW domain; HECT, homologous to the E6-AP carboxyl terminus (HECT) domain; QA, poly-glutamate and -alanine stretch (QA) domain; NLS, nuclear translocation signal; RHD, runt homology domain; NMTS, nuclear matrix targeting sequence

Then we examined whether Runx2 is a direct substrate of Wwp2-dependent ubiquitination and identified the physical interaction domains between Wwp2 and Runx2. An immunoprecipitation (IP) assay showed that overexpressed Wwp2 induced Runx2 poly-ubiquitination, which was not detectable under Wwp2-CA overexpression and was enhanced by MG132 treatment (Fig. 6a) (Supplementary Fig. 6C, D). The result of the in vitro ubiquitination assay supported the notion of Wwp2-induced Runx2 poly-ubiquitination (Fig. 6b). Consistently, Wwp2 protein, but not Wwp2-CA protein, induced Runx2 poly-ubiquitination in these in vitro assays, indicating that Runx2 is a direct substrate of Wwp2. We also confirmed the direct binding activity between Wwp2 and Runx2 by Co-IP and pull-down assay (Fig. 6c, d). ATDC5 transduced with myc-Wwp2 showed that myc-Wwp2 bound endogenous Runx2 protein (Fig. 6e). According to results of domain mapping, Runx2 bound to the WW domain of Wwp2 (Fig. 6f, g) and Wwp2 bound to the PY-motif of Runx2 protein (Fig. 6h, i).

Consequently, these data revealed that Wwp2 has an essential function to regulate Adamts5 through poly-ubiquitination-induced Runx2 degradation.

### IVT Wwp2 mRNA prevents mouse articular cartilage destruction

**Fig. 7:**
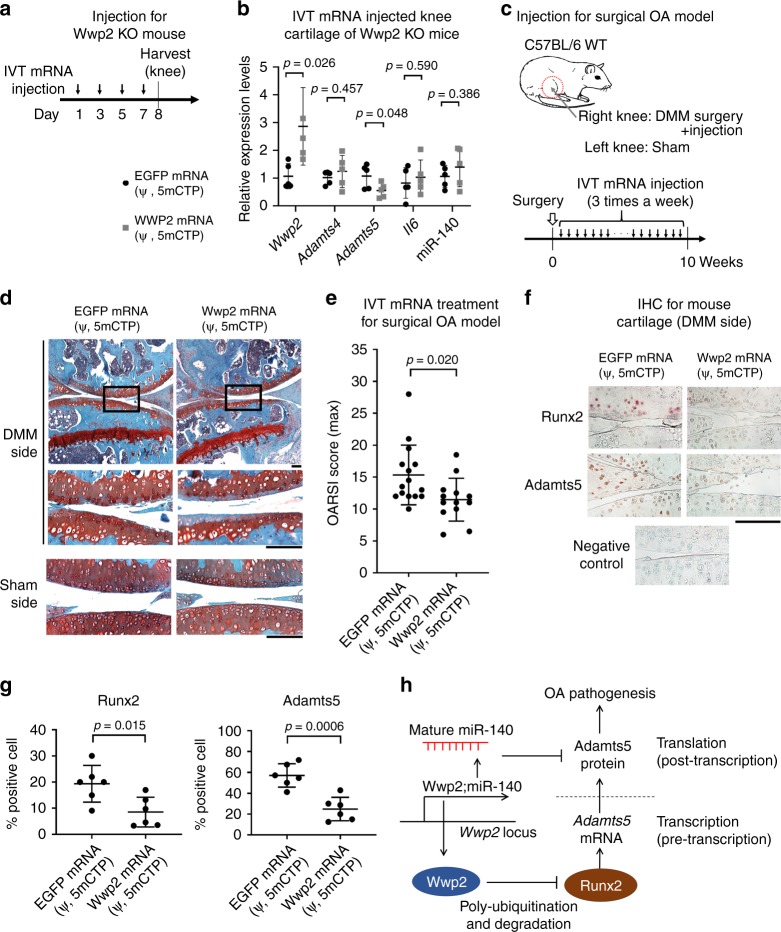
In vitro transcribed Wwp2 mRNA prevents mouse articular cartilage destruction. **a**, **b** Effect of in vivo intra-articular injection of IVT mRNA on gene expression in cartilage. IVT Wwp2 (or control EGFP) mRNAs (ψ, 5mCTP) with atelocollagen and Invivofectamine 3.0 reagent were injected into knee joints of Wwp2 KO mice. **a** Schedule of in vivo transfection. **b** RT-qPCR for OA related genes in Wwp2-transfected cartilages from injected 3-month-old Wwp2 KO mice (*n* = 5, Student’s *t* test, normalized with *Gapdh* or U6snRNA). **c**–**e** Treatment using IVT Wwp2 mRNA (ψ, 5mCTP) against surgically induced OA model in C57BL/6 mice. **c** Schedule of treatment. **d** Representative images of Safranin-O staining. **e** The maximum OARSI scores (*n* = 13-15, Student’s *t* test). **f**, **g** IHC of Runx2 and Adamts5 for IVT mRNA-treated mouse articular cartilage. **f** Representative images. Negative control was stained using rabbit control IgG. **g** Runx2 and Adamts5 positive cell rates (*n* = 6, Student’s *t* test). **h** Summarized schema of this study. Black scale bar = 1 mm. Source data are provided as a Source Data file. Data are presented as the mean ± SD

To test whether IVT Wwp2 mRNA (ψ, 5mCTP) functions in vivo, we injected IVT mRNA into knee joints of Wwp2 KO mice. After injections of IVT mRNA (ψ, 5mCTP) using a method that combines atelocollagen and invivofectamine, Wwp2 protein levels were elevated in mouse articular cartilage, as detected by IHC staining and western blot analyses, without adverse inflammatory responses or adverse injury events (Supplementary Fig. 4E, 7A, B, 8A, B, C). IVT Wwp2 mRNA (ψ, 5mCTP) significantly suppressed *Adamts5* expression levels in Wwp2 KO mice compared to IVT control mRNA (ψ, 5mCTP) (Fig. 7a, b), suggesting that introduced Wwp2 can regulate *Adamts5* expression.

On the basis of these findings, we investigated whether IVT Wwp2 mRNA (ψ, 5mCTP) has a potential therapeutic effect in the DMM OA model in mice (Fig. 7c). After DMM surgery, we injected the IVT mRNA/atelocollagen/invivofectamine mixture into C57BL/6 WT mouse knees 3 times per week for 10 weeks. Cartilage destruction in the OA model was significantly inhibited in the IVT Wwp2 mRNA-treated group compared to the control group (Fig. 7d, e) (Supplementary Fig. 2C), demonstrating the potential of Wwp2 mRNA as a new OA treatment strategy. Furthermore, the protein levels of Runx2 and Adamts5 in articular cartilage were decreased in the IVT Wwp2 mRNA-treated group compared to the control group (Fig. 7f, g) (Supplementary Fig. 2D).

In summary, Wwp2 has a protective function against cartilage destruction through regulation of Runx2-Adamts5 pathway, maintaining cartilage homeostasis. Based on the simultaneous Adamts5 targeting, this newly identified Wwp2-Runx2 pathway and previously reported miR-140, products from the *Wwp2* locus have a potential to regulate Adamts5 at pre- and post-transcriptional stages cooperatively (Fig. 7h).

## Discussion

Runx2 has dual functions; an essential role in regulating genes for intramembranous and endochondral bone development^30,31^ and catabolic action in articular cartilage by inducing matrix degradation enzymes such as Adamts5^27,28^. High *RUNX2* expression levels were detected in human OA cartilage^32^, and cartilage degradation in experimental OA was ameliorated in global Runx2-haploinsufficient mouse and conditional KO mouse (*Agc1*-CreER; *Runx2*^flox/flox^ mouse)^33,34^, supporting the notion that Runx2 contributes to OA pathogenesis. Several E3 ligases, such as Smad ubiquitination regulatory factor 1 (Smurf1), Carboxy terminus of HSP70-interacting protein (CHIP) and WW domain-containing protein 1 (Wwp1) can catalyze poly-ubiquitination of Runx2^35–37^. In osteoblasts, RUNX2 is reported to be mono-ubiquitinated by WWP2, but this does not affect protein degradation^38^. In this study, we observed that poly-ubiquitination and subsequent degradation of Runx2 was induced by Wwp2 in chondrocytes via the PY-motif of Runx2^13,25^, which is a key pathway for maintaining cartilage homeostasis.

In our study, we identified Runx2 as a target of Wwp2 and that Wwp2-Runx2 regulates Adamts5. Adamts5 is an important enzyme in OA pathogenesis. Loss of Adamts5 ameliorates cartilage degradation in murine OA models^11,12^. Importantly, it has already been reported that there are other targets of E3 ligase of Wwp2. These include TIR-domain-containing adapter-inducing interferon-β (TRIF), Notch3, Phosphatase and tensin homologue deleted on chromosome 10 (PTEN) and Src homology region 2 domain-containing phosphatase-1 (SHP-1), in addition to the Wwp2-Runx2 axis^39–42^. In this regard, in order to elucidate the molecular pathogenesis of OA triggered by Wwp2 depletion, not only Adamts5 but also other targets involved in cartilage homeostasis should be identified in future studies.

In our previous and present studies, the Wwp2 KO mouse established by the CRISPR/Cas9 system did not have developmental abnormalities, including craniofacial deformity, at 1- and 2-month-of-age. The craniofacial phenotype of *Wwp2*^GT/GT^ mouse reported by Zou et al^43^. reflects the loss of miR-140. This is not the case in the Wwp2 KO mouse, in which miR-140 expression is normal^21^. Thus, Wwp2 is not related to craniofacial deformity compared to miR-140.

Our study demonstrated that Wwp2 functions in protecting articular cartilage by regulating Adamts5. While, Wwp2 has a potential to regulate production of inflammatory cytokines in macrophages and to affect T_H_2 cells differentiation^39,42^. Experimental OA models using Wwp2 KO mouse and IVT Wwp2 mRNA (ψ, 5mCTP) treatment in the present study did not show inflammatory responses with gain or loss of Wwp2 in cartilage or synovium (Supplementary Fig. 2A, B, C) (Fig. 7b). Therefore, the inflammatory response induced by mRNA administration or gain/loss of Wwp2 does not appear to be a major mechanism explaining our results.

Administration of IVT mRNA into chondrocytes in articular cartilage in vivo should be a powerful strategy for OA treatment; however, few studies have examined this because of two major difficulties^44,45^. The efficiency of mRNA introduction in chondrocytes surrounded by ECM is low and inflammatory responses occur to introduced IVT mRNA via Toll-like receptor 3 (TLR3), Retinoic acid-inducible gene-I (RIG-I) and Melanoma differentiation-associated gene 5 (MDA5)^46^. These issues can be overcome by modifying in vivo transfection methods by combining atelocollagen and invivofectamine, and by integrating modified nucleic acids into the administrated IVT mRNA (Supplementary Fig. 7A). Modified nucleotides, including pseudouridine-5′-triphosphate (ψ) and 5-methylcytidine-5′-triphosphate (5mCTP), can modulate IVT mRNA to avoid the activation of pattern recognition receptors^23,24^ and subsequent inflammatory responses.

In cartilage homeostasis, but not during development^21^, we showed the function of Wwp2 in articular cartilage. As Adamts5 is also recognized by miR-140 (Fig. 3f), the regulation of a common target by the pair of Wwp2 and miR-140, coded in the *Wwp2* gene locus, might cooperatively enhance their function to maintain cartilage homeostasis at both pre- and post- transcriptional stages. This concept might be supported by miR-140/Wwp2-DKO mouse OA model (Fig. 1a, b) and in vitro double-overexpression experiments (Fig. 3g). Furthermore, as both miR-140 and Wwp2 are suppressed in OA cartilage and by IL-1β in chondrocytes, approaches which simultaneously induce expression of miR-140 and Wwp2 should be promising OA therapies.

## Methods

### Study approval

Human and animal studies received ethical approval by the Scripps Human Subjects Committee at The Scripps Research Institute and the Scripps Institutional Animal Care and Use Committee. Human tissues were obtained with approval by the Scripps Human Subjects Committee at The Scripps Research Institute, Protocol No: IRB-09-5162. In this study we used discarded de-identified surgical samples from Scripps Green Hospital and unidentifiable samples from tissue banks. Informed consent was not required. All animal studies were performed according to protocols approved by the Scripps Institutional Animal Care and Use Committee. All mice were freely allowed to access to food, water and activity. Quantification of histopathological changes in the joint tissues was performed by at least two independent observers blinded to the experimental conditions. Sample sizes were determined based on prior studies using wild type (WT) and mutant mice to detect similar changes in the joint tissues.

### Mice

Wwp2 KO (*Wwp2*^*1ins/1ins*^) mice, miR-140 KO (*miR-140*^14del/14del^), miR-140/Wwp2-double KO (DKO) (*Wwp2*^1del/1del^; *miR-140*^132del/132del^) and *miR-140*^-/-^ mice were generated and maintained in our lab as described^18,21^. Wwp2-C838A (Wwp2-CA) (*Wwp2*^C838A/C838A^) mice were generated based on the method described by Inui et al.^29^. Briefly, sgRNA, Cas9 mRNA and single strand oligo deoxy nucleotide (ssODN) carrying the TGC to GCC mutation were microinjected into fertilized eggs obtained from the intercross of BDF1 mice. The sgRNA used in this study was: gRNA 5′-GGCTGCCCAGGAGCCATACGTGG-3′. The synthesized ssODN (100 bases, PAGE purified) were purchased from Fasmac (Kanagawa, Japan) as follows: 5′-CTGCATCGACAGAGTTGGCAAGGAAACCTGGCTGCCCAGGAGCCATACGGCGTGAGTTTGCCGGGAGCTGGCAGGCTGGAGCTGTAGGCTGGTGGGGGCA-3′. Heterozygous mice were backcrossed to C57BL/6 mice for 4 generations before analysis.

### Micro CT analyses

To analyze the skulls and knee joints, samples were isolated from 2-month-old sex-matched wild-type (WT) and knockout (KO) mice were fixed in 99.5% ethanol and scanned using a Siemens Inveon micro-CT (Munich, Germany) scanner with a spatial resolution of 45 and 32 μm. The data were analyzed using VivoQuant software (Invicro, Boston, MA, USA).

### Surgical and aging-related mouse OA model and Wwp2 treatment

OA was surgically induced by destabilizing the medial meniscus (DMM) in the right knee joints^47^. The surgeries were performed in the right knee joints of 3-month-old male WT mice (littermate) and Wwp2 mutant mice. The left knee joints were used for sham surgeries. The knees were harvested at 10 weeks after surgery. Next, specimens were fixed in 10% zinc-formalin for 2 days and decalcified in TBD-2 (Life Technologies, Carlsbad, CA, USA) for 24 h, followed by paraffin embedding. Sections (4 µm each) from the knee joints of mice were used for Safranin-O staining and immunohistochemistry staining as described below. To observe cartilage degradation during aging, we compared male WT, Wwp2 KO, miR-140 KO and miR-140/Wwp2-DKO mice at 6, 12 and 18 months.

For the Wwp2 treatment of experimental OA, injection cocktails, containing in vitro transcribed mRNA (IVT mRNA), were prepared as described below. Male C57BL/6 WT mice had DMM surgery at 3-month-old. After DMM surgery, the IVT mRNA cocktails were injected into the right knee joints 3 times per week for 10 weeks. Knee joints were harvested and treated as described above.

### Histological analyses

Human cartilage samples for histological analyses were obtained at autopsy from individual ages 19–86 years within 48 h post-mortem under approval by the Scripps Human Subjects Committee. The articular surface from these specimens were graded macroscopically according to a modified Outerbridge scale (grade 0-4)^48^. Osteochondral blocks were harvested, fixed, decalcified, embedded and stained with Safranin-O in reference to the previous report^49^. To confirm the zonal distribution of human specimens, each zone (superficial zone, mid zone and deep zone) was classified based on previously reported characteristics^50^.

To grade mouse knee cartilage destruction of the medial femoral cartilage and medial tibial plateau, we summed the scores of the femur and tibia of Safranin-O stained samples, which were evaluated using the Osteoarthritis Research Society International (OARSI) cartilage OA histopathology semi-quantitative scoring system (score 0-24)^51^. The data are shown as maximum and average scores. Average scores were calculated using the scores of three specimens per mouse. Osteophyte, synovitis and subchondral bone were evaluated as described^52–54^.

To compare the area of the growth plate in the tibiae, the dimensions were analyzed using ImageJ 1.51 K software (NIH, Bethesda, MD, USA).

### Primary articular chondrocyte preparation and culture

Preparation of primary human articular chondrocytes, isolation was performed in reference to the previous report^55^. In brief, cartilage slices were collected from femoral condyles and washed in DMEM. After mincing with scalpels, we transferred the samples into collagenase IV (Worthington Biochemical, Lakewood, NJ, USA) contained DMEM at 37 degrees for 24 h. Isolated cells were seeded into dishes or flasks.

Primary mouse articular chondrocytes were prepared from the hips and knees of 6-days-old mice as previously reported^56^. We isolated the cartilage from the femoral heads, femoral condyles, and tibial plateau. We took care to remove hypertrophic zone in the collect tissues. Then, we treated the tissues with collagenase IV (Worthington Biochemical) in DMEM at 37 °C for 12 h. Isolated cells were seeded into dishes or flasks.

Primary chondrocytes, SW1353 cell line and HEK293T cell line (ATCC, Manassas, VA, USA) were incubated at 37 °C in humidified gas containing 5% CO_2_ with DMEM/L-glutamine/10% fetal bovine serum/penicillin-streptomycin (Life Technologies, Carlsbad, CA, USA). ATDC5 cell line (Sigma-Aldrich, St. Louis, MO, USA) was cultured in DMEM-F12/L-glutamine/HEPES/5% fetal bovine serum/penicillin-streptomycin (Life Technologies). In some experiments, primary chondrocytes and HEK293T were treated with the proteasome inhibitor MG132 (Sigma-Aldrich), poly(I:C) (InvivoGen, San Diego, CA, USA), Cycloheximide (100 ug/ml), recombinant IL-1β (R&D Systems, Minneapolis, MN, USA) (10 ng/mL) after serum starvation. IVT mRNA and microRNA mimic were transfected with Lipofectamine MessengerMAX (Life Technologies) into primary chondrocytes according to the manufacturer’s instructions. The microRNA mimic (Pre-miR miRNA Precursor of mmu-miR-140-5p and its negative control) were purchased from Life Technologies.

### Plasmid construction

Wwp2 (*Wwp2*: NM_025830.3) and RUNX2 (*Runx2*: NM_001146038.2) plasmids were constructed by inserting full-length cDNA into the pcDNA3, pcDNA3-flag, pcDNA3-myc, pcDNA3-A(124) vectors^57^ or pMXs-Neo. The Wwp2-C838A (Wwp2-CA) plasmid was constructed based on their full-length vectors by inverse PCR. Fragmented Wwp2 and Runx2 plasmids were constructed based on their full-length vectors by PCR. pcDNA3-flag-RelA (p65), pRK5-HA-Ubiquitin-WT and pRK5-EGFP vectors were purchased from Addgene (Cambridge, MA, USA).

For the luciferase assay, 5 reporter plasmids were constructed by PCR or using custom gene products (Genscript, Piscataway, NJ, USA). The pGL4.16 vector and pNL1.1 vector were purchased from Promega (Madison, WI, USA). An NF-κB reporter plasmid was constructed by inserting the HSV-TK promoter and 4 repeats of the NF-κB binding consensus sequence 5′-GGGAATTTCC-3′ into pGL4.16 vector. Runx2 reporter plasmids (6OSE2) was constructed according to a previously reported sequence^58^. Adamts5p plasmids was constructed by inserting the mouse Adamts5 promoter region (from -880 to + 67 bp relative to the TSS) into the pGL4.16 and pNL1.1 vectors.

### Generation of IVT mRNA, in vivo mRNA and siRNA transfection

To generate IVT mRNA, template plasmids based on the pcDNA3-A(124) vector were linearized, followed by in vitro transcription performed using T7 RNA polymerase enzyme from the mMESSAGE mMACHINE T7 transcription Kit (Ambion, Foster City, CA, USA). Components of the mixture except T7 polymerase, reaction buffer and template DNA were as follows: (1) conventional IVT mRNA: 6 mM Cap analog (Anti-Reverse Cap Analog), 7.5 mM ATP, 1.5 mM GTP, 7.5 mM CTP and 7.5 mM UTP. (2) IVT mRNA (ψ, 5mCTP): 10 mM Cap analog (Anti-Reverse Cap Analog), 7.5 mM ATP, 2.5 mM GTP, 7.5 mM CTP, 4 mM 5-methylcytidine-5′-triphosphate (5mCTP), 7.5 mM UTP and 4 mM pseudouridine (ψ) (TriLink BioTechnologies, San Diego, CA, USA). After in vitro transcription at 37 °C for 60 minutes, IVT mRNA was purified using the MEGAclear Kit (Ambion).

To transfect IVT mRNA into primary chondrocytes in vitro, the cells were treated with Lipofectamine MessengerMAX (Life Technologies) and IVT mRNA (ψ, 5mCTP) according to the manufacturer’s instructions. Additionally, we performed in vivo IVT mRNA transfection experiments using Invivofectamine 3.0 Reagent (Life Technologies) and AteloGene Local Use Quick Gelation (atelocollagen) (Cosmo Bio USA, Carlsbad, CA, USA). Briefly, 1 pmol IVT Wwp2 mRNA (ψ, 5mCTP), 1 pmol IVT EGFP mRNA (ψ, 5mCTP) (negative control) or 2000 ng of poly(I:C) and 3 µL Invivofectamine 3.0 Reagent were mixed, followed by incubation at 50 °C for 30 minutes. Next, 24 µL PBS, 3 µL atelocollagen and 3 µL dilution buffer were added. Also, for the in vivo siRNA transfection experiment, control siRNA (siControl) and siRNA against Runx2 (siRunx2) were purchased from Life Technologies (Ambion Silencer Select siRNA, In Vivo Ready). Briefly, 1.5 µL of 50 µM siRNA and 3 µL Invivofectamine 3.0 Reagent were mixed, followed by incubation at 50 °C for 30 minutes. Next, 18 µL PBS, 6 µL atelocollagen, and 6 µL dilution buffer were added. Finally, the reagent mixture was injected into the mouse knee at 30 µL per joint. For function evaluation of IVT mRNA, after 1 week injection (every other day) into Wwp2 KO mice, mouse knee cartilages were harvested for RT-qPCR and western blot analyses. In addition, for siRNA treatment, mouse knee cartilages were harvested after 1 week injection (every three days).

### RT-qPCR and RNA-seq for RNA expression analysis

A mouse articular cartilage was collected from the femoral condyle and tibial plateau of 2- or 3-month-old mice. Total RNA from articular cartilage and cultured cells were isolated using Zymo Direct-zol RNA MiniPrep kit (Zymo Research, Irvine, CA, USA) according to the instructions of the manufacturer. For reverse transcription, cDNA was synthesized using PrimeScript RT Reagent Kit (Takara Bio USA) and TaqMan small RNA Assays (Applied Biosystems, Foster City, CA, USA). After that, real-time PCR (RT-qPCR) was performed using LightCycler 96 System (Roche Life Science, Indianapolis, IN, USA) with custom primers and Taqman probes (Applied Biosystems), as shown in Supplementary Table 1. For RT-qPCR of microRNA, Taqman MicroRNA Assays kits (Applied Biosystems) were used, according to manufacturer’s instructions. We used the primers and Taqman probes shown in Supplementary Table 1. The data were normalized using housekeeping genes or endogenous reference genes, such as Gapdh or U6snRNA.

For RNA-seq on articular cartilage, samples were processed and sequenced on a NextSeq 500 device. To analyze the data, counts of gene expression were normalized. For human knee cartilage specimens, RNA isolation, library construction, RNA-sequencing and data analysis were performed as previously reported^59^. We extracted the data of NEDD4 family genes from our previously published data set to detect differences in Wwp2 and other NEDD4 family members in normal vs OA human articular cartilage. All analyzed data are in the Source Data file. Additionally, we also performed RNA-seq on cartilage from 2 months old WT and Wwp2 KO mice. Genes with count values ≥ 2000 and a change ≥ 1.5-log_2_fold were extracted and used for analysis. A heat map showing proteinaceous extracellular matrix related genes, which was classified by DAVID analysis. Enrichment analysis was performed using DAVID Bioinformatics Resources 6.8 (https://david.ncifcrf.gov/). The RNA-seq data is available in the NCBI Sequence Read Archive (SRA) with accession number PRJNA510523.

### Chromatin immunoprecipitation (ChIP)-qPCR

ATDC5 cells were cultured as described above and incubated in 5 plates of 15-cm culture dishes. After fixation of formaldehyde solution, sonication was performed with a Covaris S2 (Covaris, Wobrum, MA, USA). Sonicated samples were enriched by immunoprecipitation with anti-RUNX2 monoclonal antibody (host: rabbit, clone: D1L7F, Cell Signaling Technology, Danvers, MA, USA) and Dynabeads Protein A (Life Technologies). Rabbit IgG (Cell Signaling Technology) was used as a control. Input and immunoprecipitated (eluted) DNAs were analyzed by qPCR using LightCycler 96 System (Roche Life Science). Primer sequences were as follows: Adamts5 promoter (-700), FW primer 5′-ACAAAGCCAAGGACTTCCC-3′ and RV primer 5′-CCACCGGTGCTTCCTG-3′; Adamts5 promoter (-1400), FW primer 5′-CATTCAGGCTCTCTCGGACT-3′ and RV primer 5′-GAAGGCCAAACAACAGTTAAAGTAA-3′; Runx2 promoter, FW primer 5′-GTCACTACCAGCCACCG-3′ and RV primer 5′-AAAACGGAGTGAGCAAATATTTGAAG-3′; gene desert in mouse chromosome 6, FW primer 5′-ACCAAGAGCAGATCACAAAGCTA-3′ and RV primer 5′-AAATTCTGCTGTGTTCCATCATTG-3′ (Supplementary Table 4).

### Immunoprecipitation (IP) and co-IP assays

IP and co-IP experiments were performed using HEK293T cells treated with MG132 (10 μM, 7 h) (Sigma-Aldrich) and ATDC5 cells transduced with retrovirus vector. HEK293T was transfected with plasmid vectors using Lipofectamine 3000 (Life Technologies) according to manufacturer’s instructions. The cells were lysed in lysis buffer (25 mM Tris/HCl pH 7.4, 150 mM NaCl, 1% NP-40, 1 mM EDTA, 5% glycerol). Next, the samples (300 µg per sample) were incubated with primary antibodies (or control IgG), followed by incubation with Dynabeads Protein A or Protein G (Life Technologies) according to the manufacturers’ instructions. ATDC5 cells were transduced using pMXs-myc-Wwp2-transfected PLAT-A cell line (Cell Biolabs, Inc., San Diego, CA, USA) according to the manufacturer’s instructions. After washing with PBS and elution with SDS buffer, western blot analyses were performed as described below. We used the following antibodies: anti-myc tag monoclonal antibody (host: mouse, clone: 9B11, Cell Signaling Technology, 0.1 µg), anti-HA tag high-affinity monoclonal antibody (host: rat, clone 3F10, Sigma-Aldrich, 0.5 ug) and normal mouse IgG2a (Santa Cruz Biotechnology, Dallas, TX, USA, 0.1 µg).

### Ago2-crosslinking immunoprecipitation (CLIP) Analysis

CLIP was performed as previously described with modifications^60^. A total of 2 × 10^7^ chondrocytes from WT or *miR-140*^*-/-*^ mice were washed with ice-cold PBS and UV-irradiated twice with 400 and 200 mJ/cm^2^ using UV crosslinker (CL1000) (UVP, Upland, CA, USA). The cells were lysed with PXL buffer (1x PBS, 0.1% SDS, 0.5% deoxycholate, and 0.5% NP-40) on ice for 10 min, and RQ1 DNase (final conc. 30 U/mL; Promega) was added and incubated at 37 degrees for 5 minutes. After centrifugation, anti-mouse Ago2 monoclonal antibody (host: mouse, clone: 2D4, Wako Chemicals GmbH, Osaka, Japan)- or normal mouse IgG (Santa Cruz Biotechnology)-Dynabeads Protein A (Life Technologies) complex were added to the supernatants and incubated on a rotator for 2 h at 4 degrees. The Dynabeads were washed twice with PXL buffer, high salt wash buffer (5x PBS, 0.1% SDS, 0.5% deoxycholate, and 0.5% NP-40), and PNK buffer (50 mM Tris-HCl, 10 mM MgCl_2_, and 0.5% NP-40; pH 7.4), respectively, and then Proteinase K treatment, phenol-chloroform extraction, and ethanol precipitation. The precipitated-RNAs were reverse transcribed using random primer with ReverTra Ace (Toyobo Life Science Department, Osaka, Japan) according to the manufacturer’s instructions. RT-qPCR was performed using THUNDERBIRD SYBR qPCR Mix (Toyobo Life Science Department). Primer sequences were as follows: *Gapdh*, FW primer 5′-CCTGGTCACCAGGGCTGC-3′ and RV primer 5′-CGCTCCTGGAAGATGGTGATG-3′ and *Adamts5*-UTR, FW primer 5′-CACTGAAATCATCCTAAGGAGGG-3′ and RV primer 5′-CATTCCCCTGTCAATGTAGGAATA-3′ (Supplementary Table 4).

### Western blot analysis

Chondrocytes, shaved cartilage, and HEK293T cells were minced in SDS buffer. These samples and samples for IP were transferred onto polyvinylidene difluoride (PVDF) membrane after SDS-PAGE. To detect the targeted protein, we used primary antibodies as follows: anti-WWP2 polyclonal antibody (sc-11896, host: goat, Santa Cruz Biotechnology, 1:120), anti-RUNX2 monoclonal antibody (host: rabbit, clone: D1L7F, Cell Signaling Technology, 1:1000), anti-myc tag monoclonal antibody (host: mouse, clone: 9B11, Cell Signaling Technology, 1:1000), anti-myc tag monoclonal antibody (host: rabbit, clone: 71D10, Cell Signaling Technology, 1:1000), anti-DYKDDDK tag monoclonal antibody (host: mouse, clone: 9A3, Cell Signaling Technology, 1:1000), anti-HA tag polyclonal antibody (ab9110, host: rabbit, Abcam, Cambridge, MA, USA, 1:2000) and anti-GAPDH monoclonal antibody (host: rabbit, clone: 14C10, Cell Signaling Technology, 1:1000). Secondary antibody reactions and signal detection were performed using a LI-COR immunofluorescence detection system (LI-COR Biosciences, Lincoln, NE, USA). All uncropped images are provided as a Source Data File.

### Luciferase assay

SW1353 human chondrosarcoma cells were transfected with DNA using Lipofectamine 3000 (Life Technologies) according to the manufacturer’s instructions. After transfection of empty vector (mock), luciferase reporter, Wwp2, Wwp2-CA and Runx2 plasmids, the cells were incubated for 24 h. The cells were lysed and measured using Dual-Glo Luciferase Assay System (Promega) in a 96-well plate. For the NF-κB reporter plasmid, recombinant IL-1β (10 ng/mL) was added for 6 h before measurement. Cells were lysed and measured using Dual-Glo Luciferase Assay System (Promega) in a 96-well plate.

For knockdown experiments, siRNAs [siWwp2 (SI00223615, SI02743797), siRunx2 (SI00187915, SI02689526), or negative control (siControl) (1022076)] (QIAGEN, Valencia, CA, USA) were transfected into ATDC5 cells using Lipofectamine RNAiMAX (Life Technologies). Four hours after transfection, the cells were transfected with pNL1.1-6OSE2 or -Adamts5p using Fugene HD (Promega) according to the manufacturer’s instructions, and were incubated for 36 h. Cells were lysed and measured using Nano-Glo Luciferase Assay System (Promega) in 96-well plates.

### Immunohistochemistry (IHC)

IHC was performed as previously described with modifications^61^. For IHC, sections obtained from paraffin-embedded tissues were used. Following deparaffinization and antigen retrieval in citrate buffer (pH 6.0), the specimens were incubated with primary antibodies, followed by incubation with horseradish peroxidase (HRP) or alkaline phosphatase (AP)-conjugated secondary antibodies. Next, the samples were visualized using the AEC plus substrate-chromogen (Agilent Technologies, Santa Clara, CA, USA) or VECTOR Red Alkaline Phosphatase Substrate Kit (Vector Laboratories, Burlingame, CA, USA). The slides were counterstained with methyl green solution. For fluorescence IHC staining, following deparaffinization, antigen retrieval and incubation with primary antibodies, the specimens were incubated with Alexa Fluor 488 conjugated secondary antibodies and stained by DAPI (Vector Laboratories). We used the following antibodies: anti-WWP2 polyclonal antibody (sc-11896, host: goat, Santa Cruz Biotechnology, 1:50), anti-ADAMTS5 polyclonal antibody (GTX100332, host: rabbit, Genetex, Irvine, CA, USA, 1:100), anti-RUNX2 monoclonal antibody (host: rabbit, clone: EPR14334, Abcam, 1:100), normal rabbit IgG (Santa Cruz Biotechnology), ImmPRESS HRP Anti-Goat IgG (Peroxidase) Polymer Detection Kit (Vector Laboratories), ImmPRESS HRP Anti-Rabbit IgG (Peroxidase) Polymer Detection Kit (Vector Laboratories), ImmPRESS AP Anti-Rabbit IgG (alkaline phosphatase) Polymer Detection Kit and Alexa Fluor 488 conjugated anti-Goat IgG Secondary Antibody (host: donkey, Life Technologies).

### In vitro ubiquitination assay and in vitro pull-down assay

The tested proteins were obtained from plasmid-transfected HEK293T cells using tagged protein magnetic purification kit (Medical & Biological Laboratories Co., Nagoya, Japan) following the manufacturer’s instructions. The in vitro ubiquitination assay was performed using Ubiquitinylation kit (Enzo Life Science, Farmingdale, NY, USA) according to the manufacturer’s protocol. Recombinant Ubiquitin-K0 (no Lys) was purchased from R&D Systems. And, in vitro pull-down assay was performed using Dynabeads His-Tag Isolation and Pulldown (Life Technologies) following the manufacturer’s instructions.

### Statistical methods

The significance of the difference between pairs of groups was determined by unpaired *t* test (Student’s *t* test or Welch’s *t* test) after determining the variances using the F test. The differences among three or four groups were estimated using one-way analysis of variance (ANOVA) followed by Dunnett test or Kruskal–Wallis test, followed by the Dunn test, after determining the variances using Bartlett’s test. Data processing and analyses were conducted using GraphPad Prism 8 (GraphPad Software Inc., La Jolla, CA, USA). Data are presented as the mean ± SD. Results of F test and Bartlett’s test are shown in Supplementary Table 3.

### Reporting summary

Further information on research design is available in the Nature Research Reporting Summary linked to this article.

## Supplementary information

Supplementary Information

Reporting Summary

Source Data

## Data Availability

The RNA-seq data is available in the NCBI Sequence Read Archive (SRA) with accession number PRJNA510523. A reporting summary for this Article is available as a Supplementary Information file. The source data underlying most of Figures are provided as a Source Data file, except for tissue images. All relevant data are available from the authors.
