# Supplementary material for: Wwp2 maintains cartilage homeostasis through regulation of Adamts5
Source: Nat Commun. 2019 Jun 3;10:2429. doi: 10.1038/s41467-019-10177-1 (PMC6546747; doi:10.1038/s41467-019-10177-1)
Supplement: Supplementary file 1 — Supplementary Information [file 41467_2019_10177_MOESM1_ESM.pdf]

Supplementary Information

Wwp2 maintains cartilage homeostasis through regulation of Adamts5

Mokuda et al,

- Supplementary Figure 1-8
- Supplementary Table 1-4

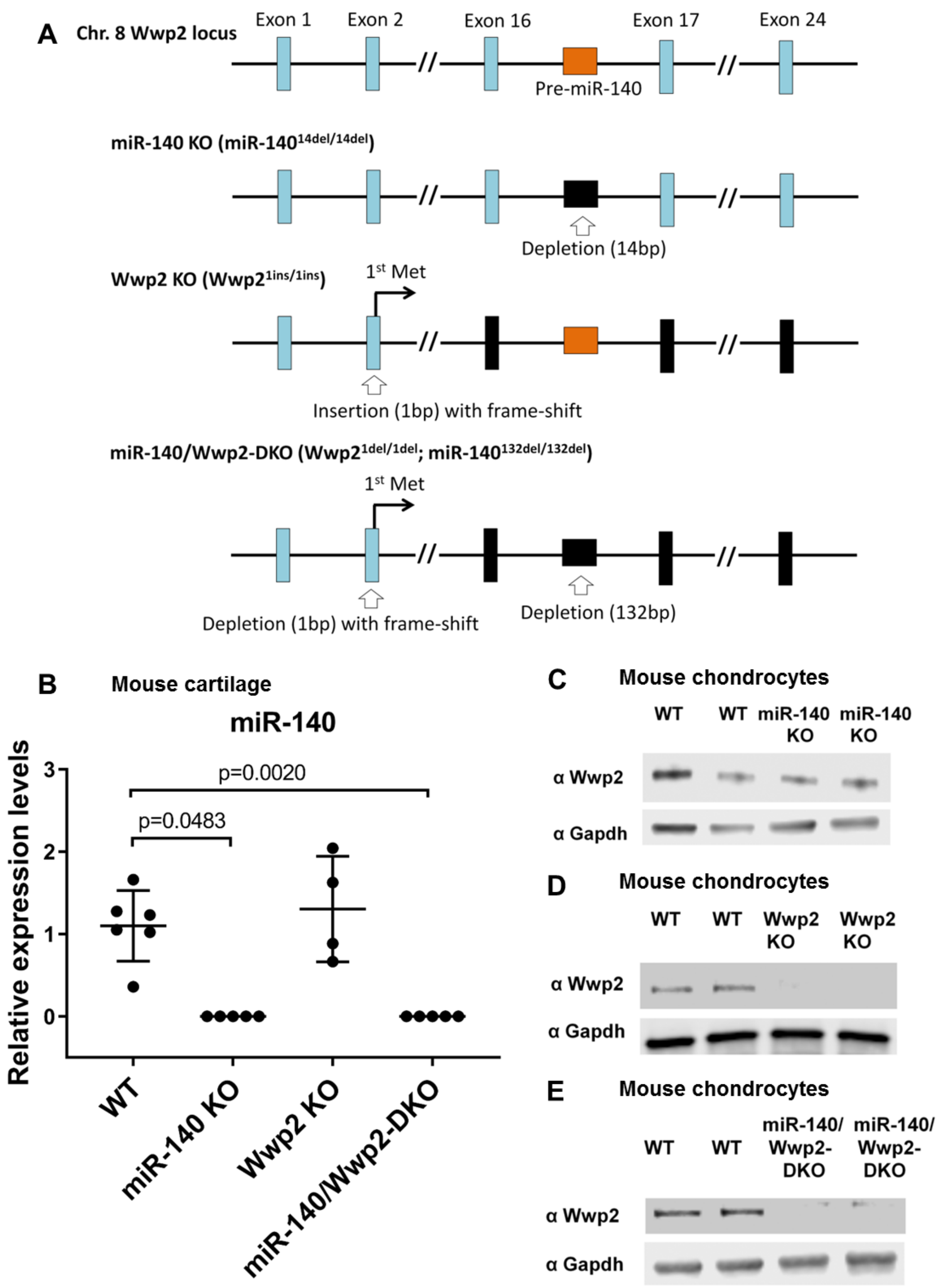

Supplementary Figure 1. miR-140 and Wwp2 expression in miR-140 KO, Wwp2 KO and miR-140/Wwp2-DKO mice

(A) Schema of *Wwp2* gene locus, which encodes miR-140 (intronic microRNA), and Wwp2 protein, and mutated *Wwp2* gene locus for miR-140 KO (*miR-140*$^{14del/14del}$), Wwp2 KO

(*Wwp2*$^{1ins/1ins}$) and miR-140/Wwp2-DKO (*Wwp2*$^{1del/1del}$; *miR-140*$^{132del/132del}$) mice. Black boxes indicate mutant products. **(B)** miR-140 expression in mouse cartilage from wild type (WT), miR-140 KO, Wwp2 KO and miR-140/Wwp2-DKO mice (n = 4-6, Dunn test compared with WT, normalized with U6snRNA). **(C-E)** Western blot analyses for mouse chondrocytes to detect Wwp2 expression. **(C)** WT and miR-140 KO. **(D)** WT and Wwp2 KO. **(E)** WT and miR-140/Wwp2-DKO. Source data are provided as a Source Data file. Data are presented as the mean ± SD.

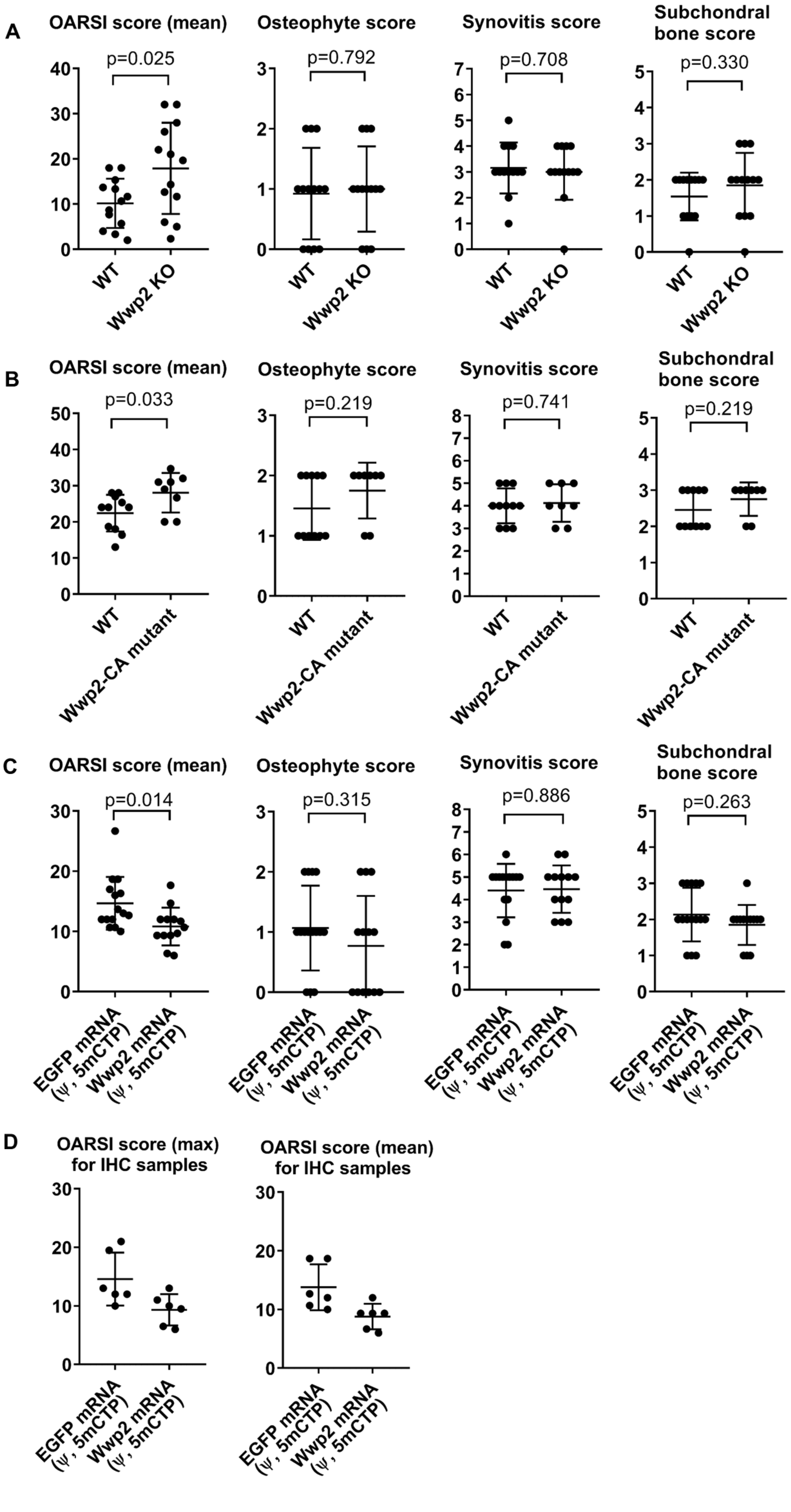
A
OARSI score (mean)
p=0.025
Osteophyte score
p=0.792
Synovitis score
p=0.708
Subchondral bone score
p=0.330
WT
Wwp2 KO
B
OARSI score (mean)
p=0.033
Osteophyte score
p=0.219
Synovitis score
p=0.741
Subchondral bone score
p=0.219
WT
Wwp2-CA mutant
C
OARSI score (mean)
p=0.014
Osteophyte score
p=0.315
Synovitis score
p=0.886
Subchondral bone score
p=0.263
EGFP mRNA (ψ, 5mCTP)
Wwp2 mRNA (ψ, 5mCTP)
D
OARSI score (max) for IHC samples
OARSI score (mean) for IHC samples
EGFP mRNA (ψ, 5mCTP)
Wwp2 mRNA (ψ, 5mCTP)

Supplementary Figure 2. Histopathology scores for surgically-induced OA model

(A-C) OARSI score (mean), osteophyte, synovitis and subchondral bone scores for the surgically induced OA model. **(A)** WT and Wwp2 KO (n = 13, Welch's t-test or Student's t-test). **(B)** WT and Wwp2-CA (*Wwp2*$^{C838A/C838A}$) mutant (n = 8-11, Student's t-test). **(C)** IVT EGFP mRNA (ψ, 5mCTP) injection and IVT Wwp2 mRNA (ψ, 5mCTP) injection (n = 13-15, Student's t-test). **(D)** OARSI scores (maximum and mean) of samples for IHC in Fig. 7g (n = 6). Source data are provided as a Source Data file. Data are presented as the mean ± SD.

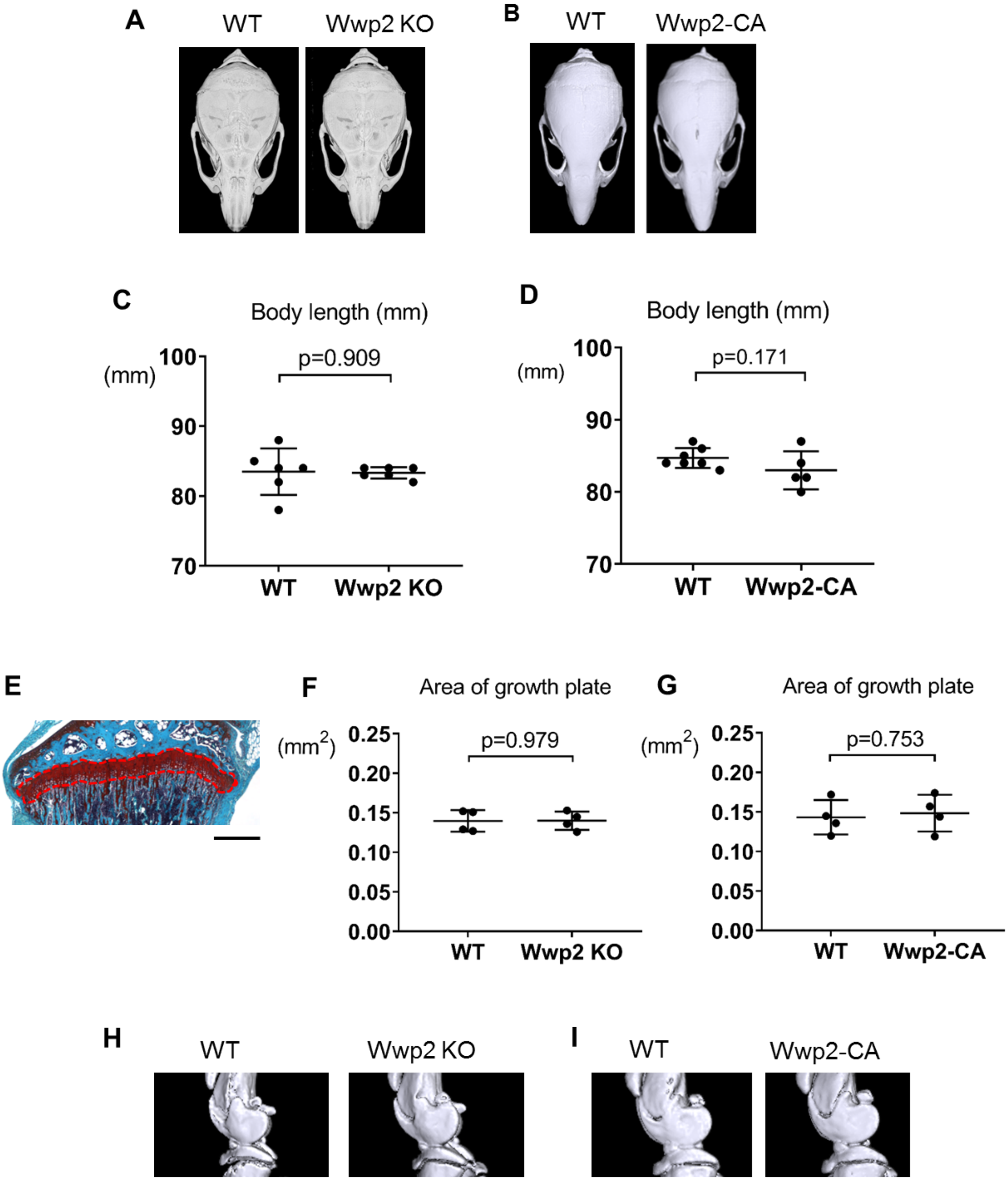

Supplementary Figure 3. Wwp2 mutant mice have no obvious developmental abnormalities

(A-B) Micro CT analyses for skulls of Wwp2 mutant mice. Skulls (2-month-old) were scanned by micro CT for WT and mutant mice, whose skulls were grossly normal. **(A)** WT and Wwp2 KO (*Wwp2*$^{1ins/1ins}$). **(B)** WT and Wwp2-CA (*Wwp2*$^{C838A/C838A}$). **(C-D)** Comparison of body length (2-month-old). **(C)** WT and Wwp2 KO (n = 6, Welch's t-test). **(D)** WT and Wwp2-CA (n = 5-7, Student's t-test). **(E-G)** Comparison of the area of the growth plate in the tibia (2-month-old). The dimensions was measured using ImageJ software. **(E)** The red dotted line indicates the traced

area. Black scale bar = 1 mm. **(F)** WT and Wwp2 KO (n = 4, Student’s t-test). **(G)** WT and Wwp2-CA (n = 4, Student’s t-test). **(H-I)** Micro CT analyses of knee joints. Right legs of 2-month-old mice were scanned by micro CT. Bone alignments from Wwp2 mutant mice were grossly normal. **(H)** WT and Wwp2 KO. **(I)** WT and Wwp2-CA. Source data are provided as a Source Data file. Data are presented as the mean ± SD.

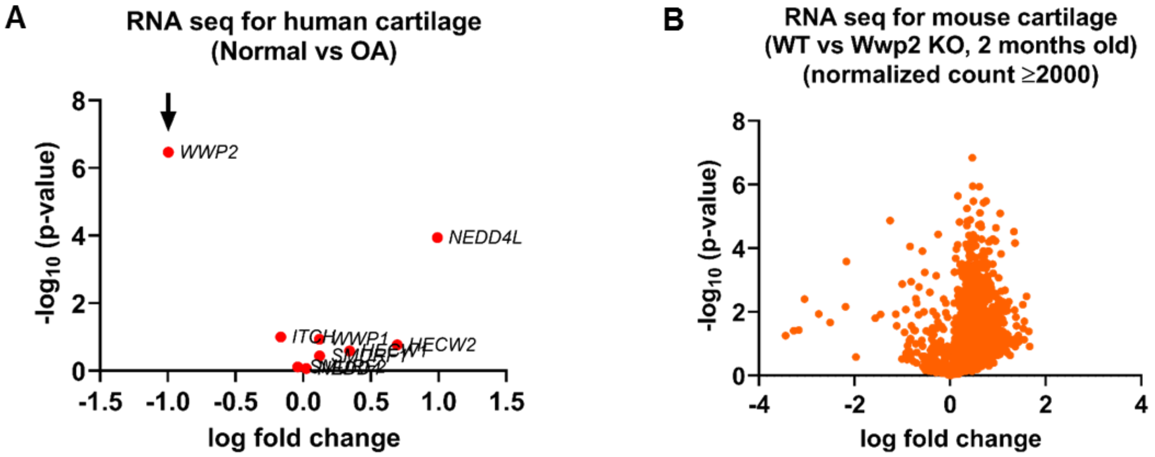

C Top-regulated genes

TOP 10 up-regulated	TOP 10 down-regulated
Tubb2a	C3
Ppp1r3c	Fgl2
Slc39a14	Trf
Nampt	Nr1d1
Hapln1	Fasn
Cytl1	Cd36
Sgk3	Car3
Stk26	Lpl
Malat1	Scd1
Meg3	Fabp4

D Top gene ontology pathways

Enrichment Score: 15.44		
Group	p-value	Number of Genes
Glycoprotein	7.7E-25	180
Signal peptide	5.1E-21	168
proteinaceous extracellular matrix	4.7E-17	34
Glycosylation site: N-linked	6.4E-15	168
extracellular region	4.2E-14	95
extracellular space	1.4E-13	84
disulfide bond	8.2E-8	108

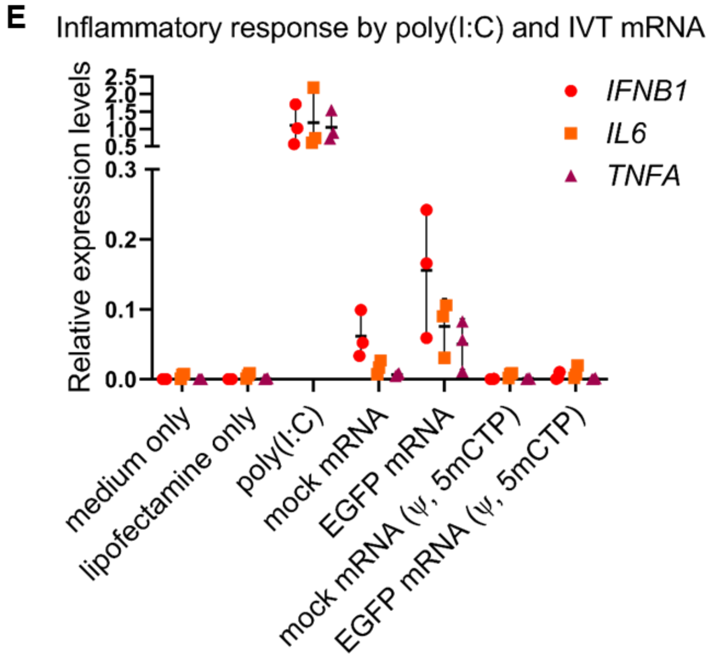

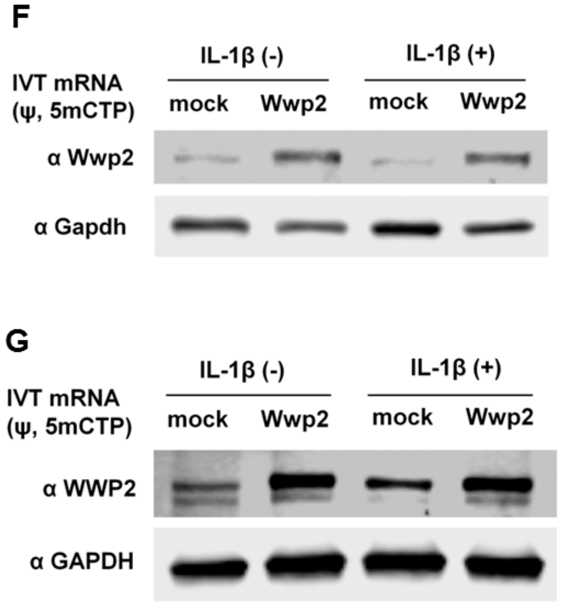

Supplementary Figure 4. RNA-seq data from human and mouse cartilages and IVT mRNA transfection of chondrocytes *in vitro* (inflammatory response and efficiency)

(A) Volcano plots for NEDD4 family proteins in human articular cartilages (Normal vs OA). Arrow indicates the WWP2 plot. **(B-D)** RNA-seq for mouse articular cartilages (WT vs Wwp2 KO

mice). **(B)** Volcano plots (normalized count values ≥2000). **(C)** List of top-regulated genes. **(D)** List of top gene ontology pathways examined by DAVID analysis. **(E)** Human chondrocytes were stimulated by Lipofectamine MessangerMAX and 0.5 pmol of *in vitro* transcribed (IVT) mRNA (or 500 ng of poly(I:C) for the positive control) for 24 hours. The data showed that modified nucleic acids (pseudouridine-5'-triphosphate (ψ) and 5-methylcytidine-5'-triphosphate (5mCTP)) reduced inflammatory response against single-stranded RNA, detected by RT-qPCR (n = 3, normalized with *GAPDH*). **(F-G)** Western blot analyses showed that Wwp2 mRNA (ψ, 5mCTP) *in vitro* transfection into chondrocytes increased Wwp2 protein levels. **(F)** Wild type (WT) mouse chondrocytes. **(G)** Human chondrocytes. Source data are provided as a Source Data file. Data are presented as the mean ± SD.

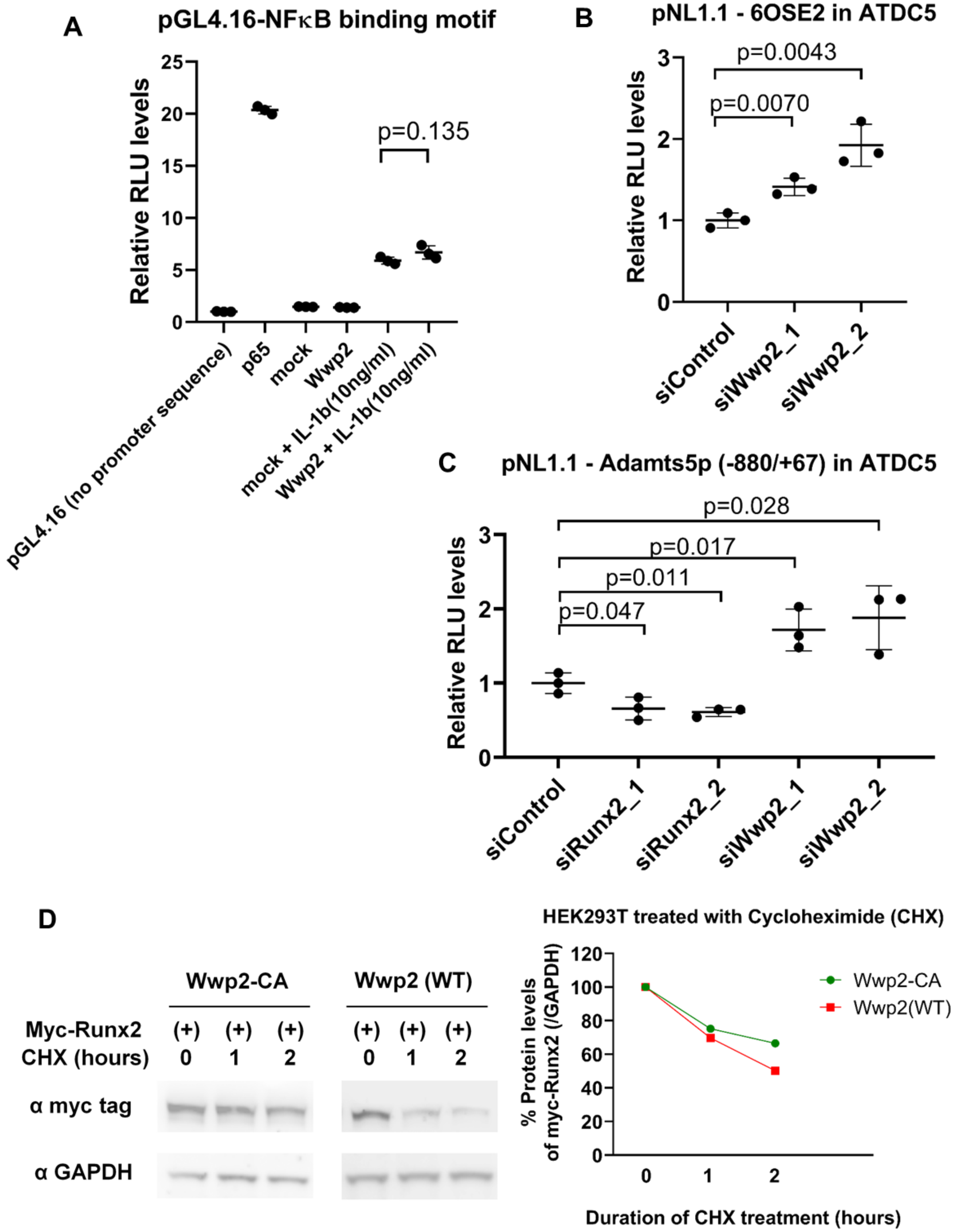

Supplementary Figure 5. Supportive data for suppressive effect of Wwp2 on Runx2 and Adamts5

(A) SW1353 cells were transfected with plasmids and stimulated with IL-1β (10 ng/mL) for 6 hours (n=3, Student’s t-test). Overexpression of Wwp2 did not affect NF-κB reporter luciferase

activities. **(B,C)** Luciferase assays using ATDC5 treated with siRNA and pNL1.1 plasmids. **(B)** pNL1.1-6OSE2 (n=3, Student's t-test). siWwp2; siRNA against Wwp2. **(C)** pNL1.1-Adamts5 promoter (n=3, Student's t-test). siRunx2; siRNA against Runx2. **(D)** Cycloheximide (CHX) blockade to detect Runx2 degradation induced by Wwp2. HEK293T cells was transfected with Wwp2 (WT) (or Wwp2-CA control) and myc-Runx2. Then, the cells were treated with CHX 100 mg/L for 2 hours. Cell lysates were blotted with anti-myc antibody. Runx2 protein stability modified with Wwp2 (WT) was degraded, compared with Wwp2-CA control. Source data are provided as a Source Data file. Data are presented as the mean ± SD.

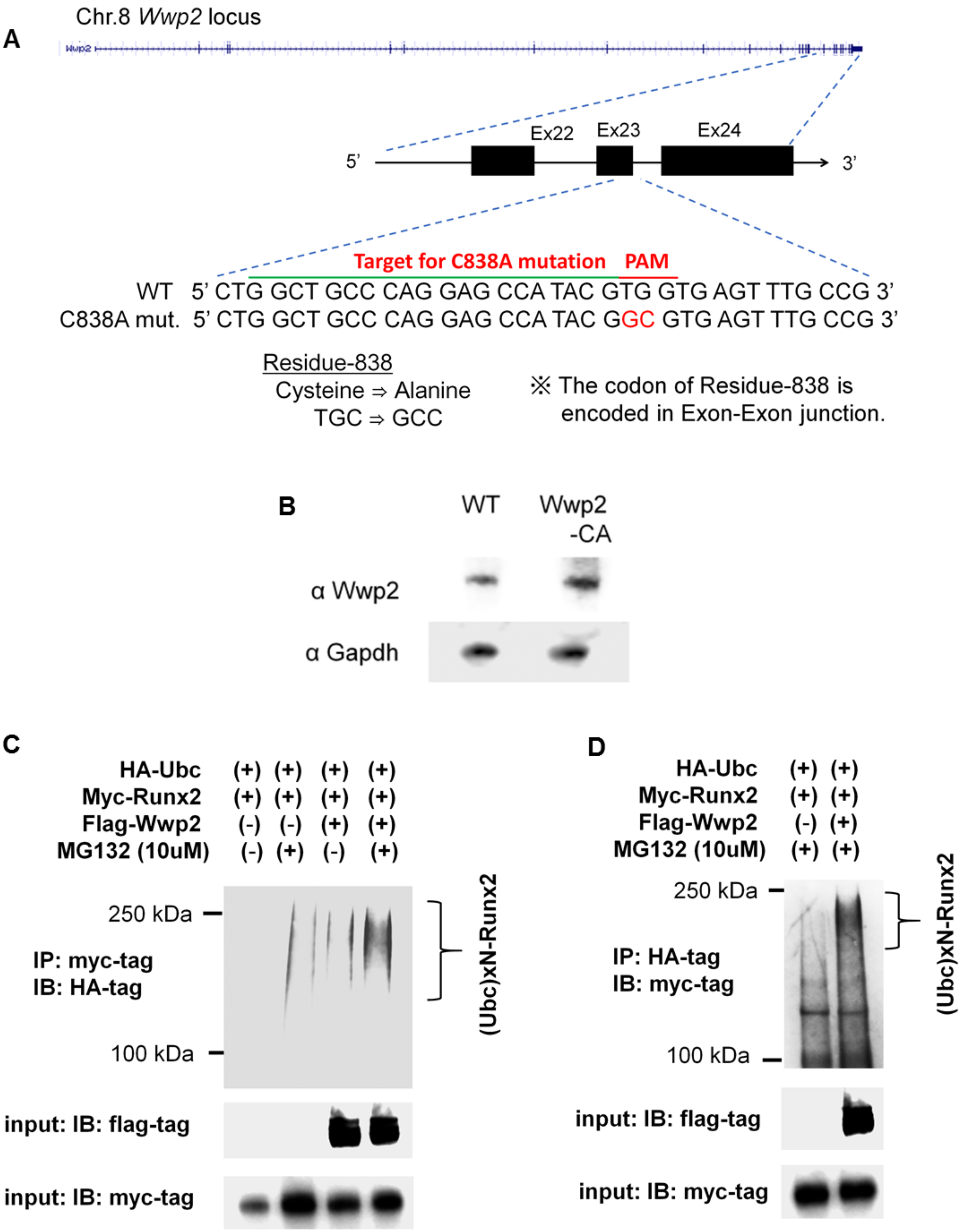

Supplementary Figure 6. Design of Wwp2-CA mutant mouse

(A) We generated Wwp2-CA (*Wwp2*$^{C838A/C838A}$) mutant mouse using the CRISPR/Cas9 system.

The sequences of sgRNA and synthesized ssDNA donor are described in the Methods. **(B)** Western blot analyses for cartilage of Wwp2-CA mice. Western blot analysis showed that Wwp2 protein was detectable in Wwp2-CA (*Wwp2*$^{C838A/C838A}$) mouse cartilage, similar to WT. **(C)** MG132 increased the protein level of poly-ubiquitinated Runx2. HEK293T cells were transfected with plasmids (HA-ubiquitin (Ubc), myc -Runx2, flag-Wwp2) and were untreated or treated with MG132 (10 μM, 7 hours). The cell lysates were treated by anti-myc-tag antibody and protein A for IP. **(D)** To detect poly-ubiquitinated Runx2 using anti-myc-tag antibody, IP was performed. The cell lysates were treated by anti-HA-tag antibody and protein G for IP. Source data are provided as a Source Data file.

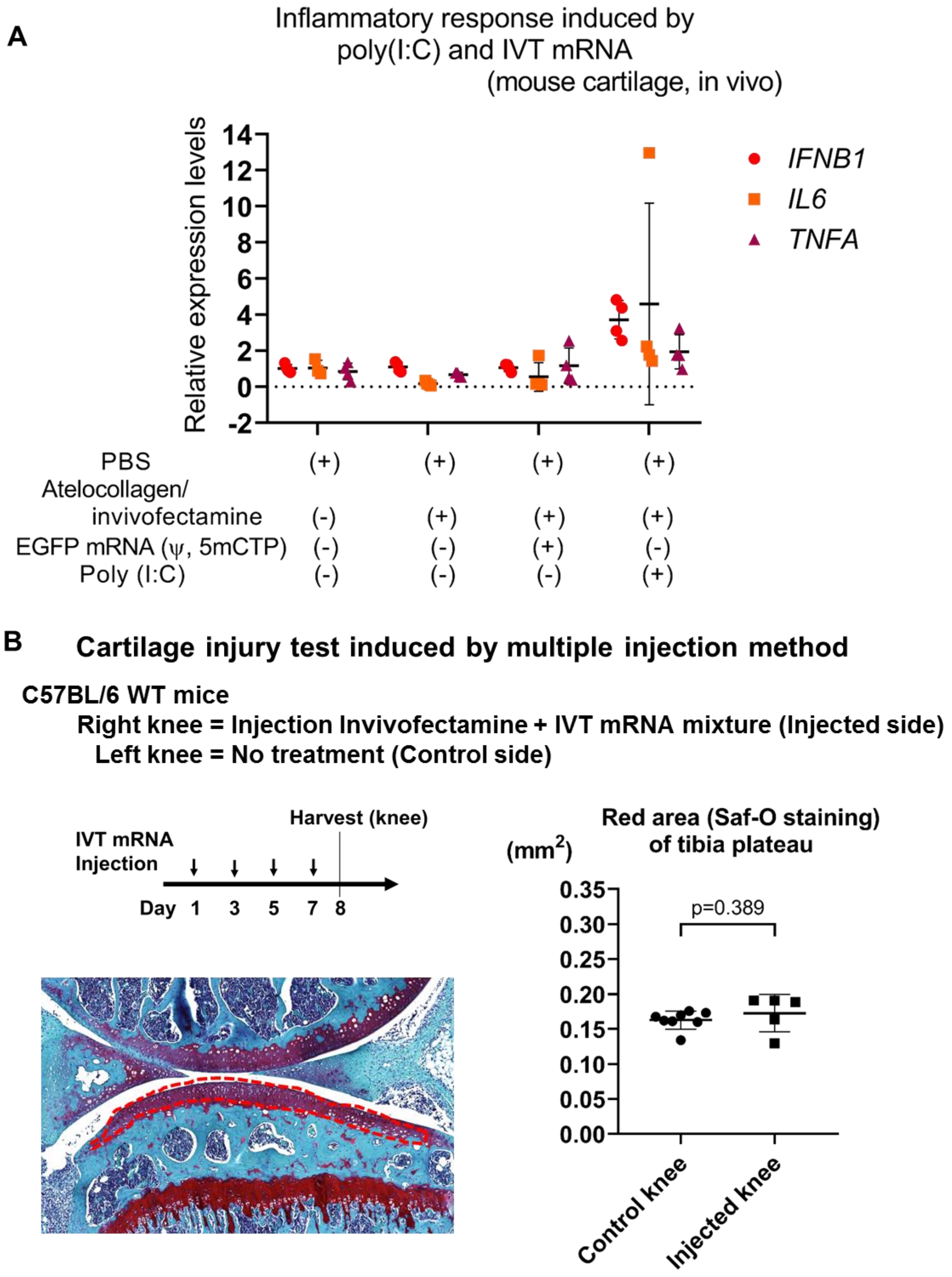

Supplementary Figure 7. Treatment of IVT mRNAs containing modified nucleic acids for mouse articular cartilage *in vivo* does not induce inflammatory response

(A) Mouse articular cartilages from wild type (WT) mice were treated with atelocollagen, Invivofectamine 3.0 and 1.0 pmol of *in vitro* transcribed (IVT) EGFP mRNA (or 2000 ng of poly(I:C)

as positive control) *in vivo*. After injections, the mouse knees were harvested for RT-qPCR (n = 4, normalized with *Gapdh*). This data showed that IVT mRNA containing modified nucleic acids (pseudouridine-5'-triphosphate (ψ) and 5-methylcytidine-5'-triphosphate (5mCTP)) do not induce inflammatory responses. **(B)** To detect whether cartilage injury was induced by multiple injection, the area of the tibia plateau was measured (red dotted line) after four injections (n = 5-8, Student's t-test). Source data are provided as a Source Data file. Data are presented as the mean ± SD.

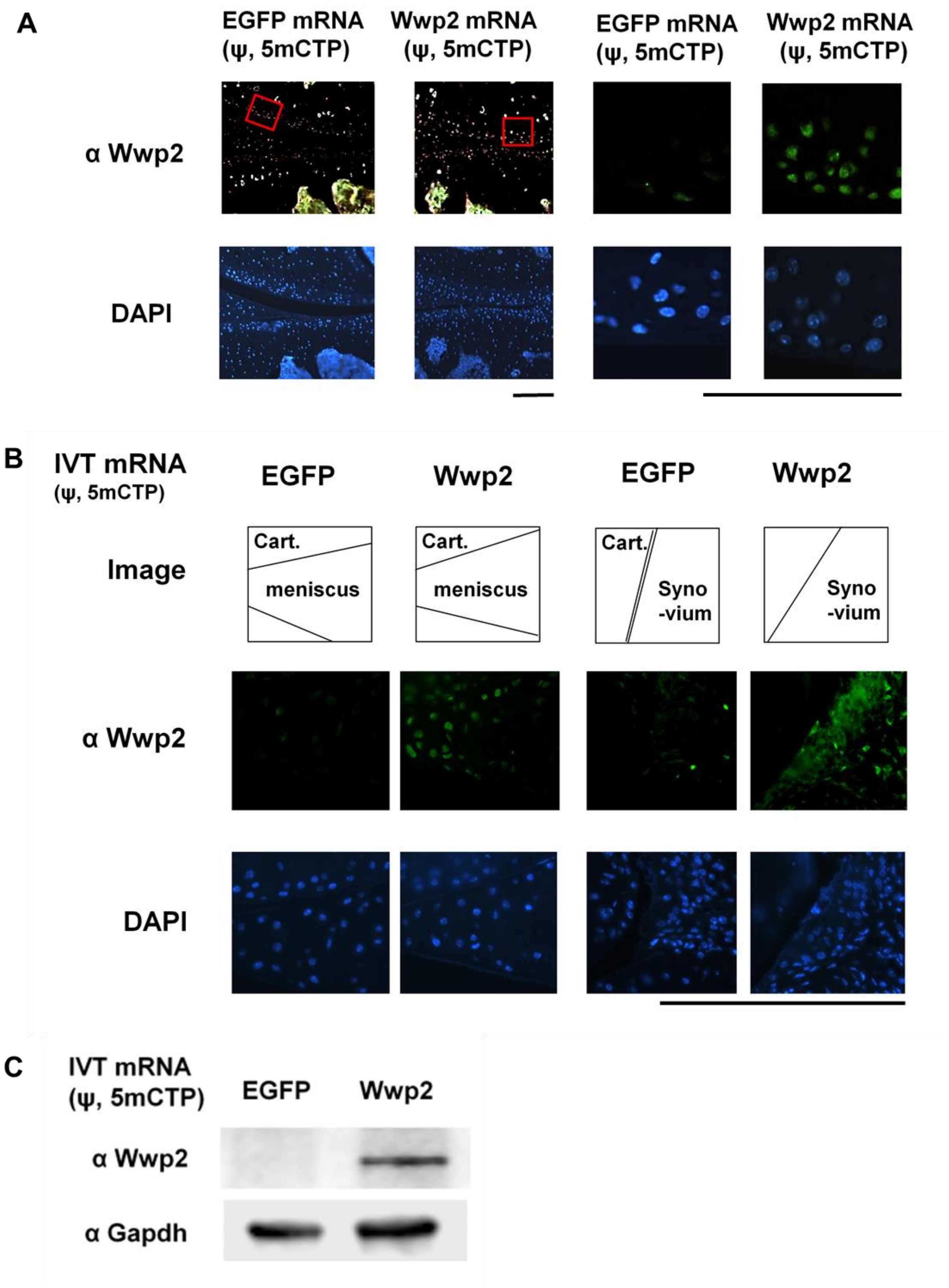

Supplementary Figure 8. Efficiency of IVT mRNA (ψ, 5mCTP) transduction of mouse articular cartilage *in vivo*

(A-B) IVT Wwp2 mRNA (ψ, 5mCTP) introduction for articular cartilage, meniscus and synovium were detected by IHC. Wwp2 was stained with Alexa Flour 488 (green). Nuclei were

labeled with DAPI (blue). **(A)** Articular cartilage. **(B)** Meniscus and synovium. **(C)** Western blot analysis showed that Wwp2 mRNA (ψ, 5mCTP) in vivo transfection into Wwp2 KO mice induced Wwp2 protein production in articular cartilage. Black Scale bar = 1mm. Source data are provided as a Source Data file.

Supplementary Table 1. List of TaqMan probes

Species / Gene symbol	Probe ID (from Applied Biosystems)
human *WWP2*	Hs00941273_m1
human *ADAMTS5*	Hs00199841_m1
human *IFNB1*	Hs01077958_s1
human *IL6*	Hs00985639_m1
human *TNFA*	Hs00174128_m1
human *GAPDH*	Hs02758991_g1
mouse *Wwp2*	Mm00627908_m1
mouse *Adamts5*	Mm01344182_m1
mouse *Adamts4*	Mm00556068_m1
mouse *Ifnb1*	Mm00439552_s1
mouse *Tnfa*	Mm00443258_m1
mouse *Il6*	Mm00446190_m1
mouse *Gapdh*	Mm99999915_g1
mouse miR-140	001187 (Taqman MicroRNA Assays)
mouse U6snRNA	001973 (Taqman MicroRNA Assays)

Supplementary Table 2. List of human subjects

(M = Male, F = Female)

A. Figure 2a

Subjects	Age	Sex	Use in different experiments
Normal cart. 1	31	M	No
Normal cart. 2	39	F	No
Normal cart. 3	52	F	No
Normal cart. 4	27	F	No
Normal cart. 5	33	M	No
Normal cart. 6	56	M	No
Normal cart. 7	18	M	No
Normal cart. 8	46	M	No
OA cart. 1	52	F	No
OA cart. 2	57	F	No
OA cart. 3	67	F	No
OA cart. 4	60	M	No
OA cart. 5	51	M	No
OA cart. 6	67	M	No
OA cart. 7	62	F	No
OA cart. 8	69	F	No
OA cart. 9	71	F	No
OA cart. 10	71	F	No

B. Figure 2b

Subjects	Age	Sex	Use in different experiments
Normal cart. 1	31	M	No
Normal cart. 2	62	M	No
Normal cart. 3	56	F	No
Normal cart. 4	57	M	No
Normal cart. 5	46	F	No
Normal cart. 6	58	M	No
Normal cart. 7	58	F	No
Normal cart. 8	36	M	No
OA cart. 1	52	F	No
OA cart. 2	74	M	No
OA cart. 3	74	F	No
OA cart. 4	57	F	No
OA cart. 5	67	F	No
OA cart. 6	60	M	No
OA cart. 7	69	F	No
OA cart. 8	61	F	No
OA cart. 9	64	F	No
OA cart. 10	51	M	No

C. Figure 2e

Subjects	Age	Sex	Use in different experiments
grade 0. 1	18	M	No
grade 0. 2	17	F	No
grade 0. 3	19	M	No
grade 0. 4	21	F	No
grade 0. 5	21	F	No
grade 0. 6	18	M	No
grade 0. 7	17	F	No
grade I. 1	41	M	No
grade I. 2	40	M	No
grade I. 3	68	F	No
grade I. 4	48	F	No
grade I. 5	76	M	No
grade I. 6	23	F	No
grade I. 7	36	F	No
grade I. 8	26	M	No
grade III. 1	80	M	No
grade III. 2	84	F	No
grade III. 3	90	F	No
grade III. 4	64	M	No
grade III. 5	88	F	No
grade III. 6	52	M	No
grade III. 7	38	F	No

Supplementary Table 3. List of F test, Bartlett test and 95%CI

Figure	Item	F test or Bartlett test (p-value)	Testing	95% confidence interval
Supplementary Figure 1B	-	< 0.001	Dunn	N/A
Supplementary Figure 2A	OARSI score (mean)	0.041	Welch	1.071 to 14.42
	Osteophyte	0.808	Student	-0.5171 to 0.6710
	Synovitis	0.760	Student	-0.9914 to 0.6837
	Subchondral bone	0.299	Student	-0.3307 to 0.9460
Supplementary Figure 2B	OARSI score (mean)	0.807	Student	0.5049 to 10.79
	Osteophyte	0.771	Student	-0.1934 to 0.7843
	Synovitis	0.803	Student	-0.6591 to 0.9091
	Subchondral bone	0.771	Student	-0.1934 to 0.7843
Supplementary Figure 2C	OARSI score (mean)	0.251	Student	-6.850 to -0.8418
	Osteophyte	0.544	Student	-0.8938 to 0.2989
	Synovitis	0.686	Student	-0.8137 to 0.9368
	Subchondral bone	0.316	Student	-0.8035 to 0.2292
Figure 1b	-	0.011	Dunn	N/A
Figure 1d	-	< 0.001	Welch	0.5774 to 15.06
Figure 1f	-	0.057	Student	0.7947 to 13.97
Supplementary Figure 3C	-	0.008	Welch	-3.654 to 3.320
Supplementary Figure 3D	-	0.153	Student	-4.305 to 0.8763
Supplementary Figure 3F	-	0.804	Student	-0.02164 to 0.02214
Supplementary Figure 3G	-	0.920	Student	-0.0337 to 0.0442
Figure 2a	*WWP2*	0.009	Welch	-16320 to -2025
Figure 2b	-	0.001	Welch	-1.082 to -0.07422
Figure 2e	SZ	0.608	Dunn	N/A
	MZ	0.025	Dunn	N/A
	DZ	0.040	Dunn	N/A
Figure 2g	-	0.032	Welch	-65.46 to -1.976
Figure 2h	Human	0.426	Student	-0.3845 to -0.002240
	Mouse (WT)	0.551	Student	-0.7671 to -0.2586
Figure 3b	-	0.888	Student	0.01959 to 1.265
Figure 3d	-	0.808	Student	0.1296 to 43.65
Figure 3e	Mouse (WT) (1) vs (2)	0.262	Student	-0.7352 to -0.1046
	Mouse (WT) (3) vs (4)	0.716	Student	-2.852 to -0.0002712
	Human (3) vs (4)	0.323	Student	-5.749 to -1.128
Figure 3f	*Adamts5*	0.013	Welch	-9.091 to -0.1116
Figure 3g	-	0.407	Dunnett	N/A
Supplementary Figure 5A	-	0.439	Student	-0.3773 to 1.932
Supplementary Figure 5B	vs siWwp2_1	0.841	Student	0.1881 to 0.6379
	vs siWwp2_2	0.212	Student	0.4828 to 1.363
Supplementary Figure 5C	vs siRunx2_1	0.897	Student	-0.6740 to -0.008633
	vs siRunx2_2	0.317	Student	-0.6312 to -0.1454
	vs siWwp2_1	0.394	Student	0.2152 to 1.219
	vs siWwp2_2	0.189	Student	0.1572 to 1.606
Figure 4a	-	0.862	Student	-3.830 to -2.610
Figure 4d	-	0.020	Welch	9.199 to 36.39
Figure 4e	-	0.853	Student	-0.1478 to -0.06424
Figure 4f	-	0.053	Student	-2.808 to -0.01311
Figure 4g	Adamts5 promoter (-700)	0.039	Welch	0.1668 to 2.095
	Runx2 promoter	0.135	Welch	0.3640 to 1.497
Figure 4i	-	< 0.001	Dunn	N/A
Figure 5c	-	0.448	Student	0.04366 to 10.75
Figure 5d	*Adamts5*	< 0.001	Welch	0.4509 to 4.736
	miR-140	0.545	Welch	-0.4485 to 1.023
Figure 5f	Runx2	0.024	Welch	5.404 to 30.27
	Adamts5	0.447	Welch	6.787 to 33.49
Figure 7b	*Wwp2*	0.051	Student	0.2832 to 3.307
	Adamts4	0.107	Student	-0.4269 to 0.8645
	Adamts5	0.337	Student	-1.033 to -0.006310
	Il6	0.821	Student	-0.6463 to 1.062
	miR-140	0.297	Student	-0.5019 to 1.164
Figure 7e	-	0.256	Student	-7.082 to -0.6619
Figure 7g	Runx2	0.658	Student	-19.00 to -2.574
	Adamts5	0.999	Student	-46.74 to -17.69
Supplementary Figure 7B	-	0.096	Student	-0.01420 to 0.03377

Supplementary Table 4. List of primers

Name	Sequence(5'-3')	Species
Adamts5 promoter (-700) FW	ACAAAGCCAAGGACTTCCC	Mus musculus
Adamts5 promoter (-700) RV	CCACCGGTGCTTCCTG	Mus musculus
Adamts5 promoter (-1400) FW	CATTCAGGCTCTCTCGGACT	Mus musculus
Adamts5 promoter (-1400) RV	GAAGGCCAAACAACAGTTAAAGTAA	Mus musculus
Runx2 promoter FW	GTCACTACCAGCCACCG	Mus musculus
Runx2 promoter RV	AAAACGGAGTGAGCAAATATTTGAAG	Mus musculus
Gene desert FW	ACCAAGAGCAGATCACAAAGCTA	Mus musculus
Gene desert RV	AAATTCTGCTGTGTTCCATCATTG	Mus musculus
Gapdh FW	CCTGGTCACCAGGGCTGC	Mus musculus
Gapdh RV	CGCTCCTGGAAGATGGTGATG	Mus musculus
Adamts5-UTR FW	CACTGAAATCATCCTAAGGAGGG	Mus musculus
Adamts5-UTR RV	CATTCCCCTGTCAATGTAGGAATA	Mus musculus
