# Supplementary material for: Wwp2 maintains cartilage homeostasis through regulation of Adamts5
Source: Nat Commun. 2019 Jun 3;10:2429. doi: 10.1038/s41467-019-10177-1 (PMC6546747; doi:10.1038/s41467-019-10177-1)
Supplement: Supplementary file 2 — Reporting Summary [file 41467_2019_10177_MOESM2_ESM.pdf]

nature research

Corresponding author(s): Hiroshi Asahara

Reporting Summary

Nature Research wishes to improve the reproducibility of the work that we publish. This form provides structure for consistency and transparency in reporting. For further information on Nature Research policies, see Authors & Referees and the Editorial Policy Checklist.

Statistical parameters

When statistical analyses are reported, confirm that the following items are present in the relevant location (e.g. figure legend, table legend, main text, or Methods section).

n/a	Confirmed	
☐	☒	The exact sample size (*n*) for each experimental group/condition, given as a discrete number and unit of measurement
☐	☒	An indication of whether measurements were taken from distinct samples or whether the same sample was measured repeatedly
☐	☒	The statistical test(s) used AND whether they are one- or two-sided<br>*Only common tests should be described solely by name; describe more complex techniques in the Methods section.*
☒	☐	A description of all covariates tested
☐	☒	A description of any assumptions or corrections, such as tests of normality and adjustment for multiple comparisons
☐	☒	A full description of the statistics including central tendency (e.g. means) or other basic estimates (e.g. regression coefficient) AND variation (e.g. standard deviation) or associated estimates of uncertainty (e.g. confidence intervals)
☐	☒	For null hypothesis testing, the test statistic (e.g. *F*, *t*, *r*) with confidence intervals, effect sizes, degrees of freedom and *P* value noted<br>*Give P values as exact values whenever suitable.*
☒	☐	For Bayesian analysis, information on the choice of priors and Markov chain Monte Carlo settings
☒	☐	For hierarchical and complex designs, identification of the appropriate level for tests and full reporting of outcomes
☒	☐	Estimates of effect sizes (e.g. Cohen's *d*, Pearson's *r*), indicating how they were calculated
☐	☒	Clearly defined error bars<br>*State explicitly what error bars represent (e.g. SD, SE, CI)*

Our web collection on statistics for biologists may be useful.

Software and code

Policy information about availability of computer code

Data collection	N/A
Data analysis	Statistical analysis was performed using GraphPad Prism 8. RNA-seq and ChIP-seq data was initially analyzed using edge R, followed by GraphPad Prism 8. Histological measurement was analyzed using Image J. Immunoblot was analyzed Li-Cor Image Studio Lite.

For manuscripts utilizing custom algorithms or software that are central to the research but not yet described in published literature, software must be made available to editors/reviewers upon request. We strongly encourage code deposition in a community repository (e.g. GitHub). See the Nature Research guidelines for submitting code & software for further information.

Data

Policy information about availability of data

All manuscripts must include a data availability statement. This statement should provide the following information, where applicable:

- Accession codes, unique identifiers, or web links for publicly available datasets
- A list of figures that have associated raw data
- A description of any restrictions on data availability

The source data that support the findings of this study are available from the corresponding author upon request.
RNA-seq and ChIP-seq data are available in the NCBI Sequence Read Archive (SRA) with accession number PRJNA510523.

Field-specific reporting

Please select the best fit for your research. If you are not sure, read the appropriate sections before making your selection.

☒ Life sciences ☐ Behavioural & social sciences ☐ Ecological, evolutionary & environmental sciences

For a reference copy of the document with all sections, see nature.com/authors/policies/ReportingSummary-flat.pdf

Life sciences study design

All studies must disclose on these points even when the disclosure is negative.

Sample size	No sample size calculation was performed.
Data exclusions	We used mice which were within 18 months old. Mice older 18 months were excluded from this study.
Replication	Each experiment was repeated at least twice as indicated in the figure legend. All experiment data was reproducible with similar observation in each attempt.
Randomization	No randomization was used in this experiments. Experimental groups were based on age-sex-match human subjects or genotypes of age-match mice in each experiment.
Blinding	Quantification of histopathological changes in the joint tissues was performed by at least two independent observers blinded to the experimental conditions.

Reporting for specific materials, systems and methods

Materials & experimental systems

n/a	Involved in the study	
☒	☐	Unique biological materials
☐	☒	Antibodies
☐	☒	Eukaryotic cell lines
☒	☐	Palaeontology
☐	☒	Animals and other organisms
☐	☒	Human research participants

Methods

n/a	Involved in the study	
☐	☒	ChIP-seq
☒	☐	Flow cytometry
☒	☐	MRI-based neuroimaging

Antibodies

Antibodies used	ChIP: anti-RUNX2 monoclonal antibody (host: rabbit, clone: D1L7F, Cell Signaling Technology, Danvers, MA, USA), IP: anti-myc tag monoclonal antibody (host: mouse, clone: 9B11, Cell Signaling Technology, 0.1 µg), anti-HA tag high affinity monoclonal antibody (host: rat, clone 3F10, Sigma-Aldrich, 0.5 ug), normal mouse IgG2a (Santa Cruz Biotechnology, Dallas, TX, USA, 0.1 µg), CLIP: anti-mouse Ago2 monoclonal antibody (host: mouse, clone: 2D4, Wako Chemicals GmbH, Osaka, Japan), normal mouse IgG (Santa Cruz Biotechnology), WB (primary Ab): anti-WWP2 polyclonal antibody (sc-11896, host: goat, Santa Cruz Biotechnology, 1:120), anti-RUNX2 monoclonal antibody (host: rabbit, clone: D1L7F, Cell Signaling Technology, 1:1000), anti-myc tag monoclonal antibody (host: mouse, clone: 9B11, Cell Signaling Technology, 1:1000), anti-myc tag monoclonal antibody (host: rabbit, clone: 71D10, Cell Signaling Technology, 1:1000), anti-DYKDDDK tag monoclonal antibody (host: mouse, clone: 9A3, Cell Signaling Technology, 1:1000), anti-HA tag polyclonal antibody (ab9110, host: rabbit, Abcam, Cambridge, MA, USA, 1:2000), anti-GAPDH monoclonal antibody (host: rabbit, clone: 14C10, Cell Signaling Technology, 1:1000). WB (Secondary Ab): LI-COR immunofluorescence detection system (LI-COR Biosciences, Lincoln, NE, USA). IHC: anti-WWP2 polyclonal antibody (sc-11896, host: goat, Santa Cruz Biotechnology, 1:50), anti-ADAMTS5 polyclonal antibody (GTX100332, host: rabbit, Genetex, Irvine, CA, USA, 1:100), anti-RUNX2 monoclonal antibody (host: rabbit, clone: EPR14334, Abcam, 1:100), normal rabbit IgG (Santa Cruz Biotechnology), ImmPRESS HRP Anti-Goat IgG (Peroxidase) Polymer Detection Kit (Vector Laboratories), ImmPRESS HRP Anti-Rabbit IgG (Peroxidase) Polymer Detection Kit (Vector Laboratories), ImmPRESS AP Anti-Rabbit IgG (alkaline phosphatase) Polymer Detection Kit and Alexa Fluor 488 conjugated anti-Goat IgG Secondary Antibody (host: donkey, Life Technologies).
Validation	All antibody used in this study were obtained from commercial sources and validated according to manufacturers's instruction.

Eukaryotic cell lines

Policy information about cell lines

Cell line source(s)	HEK293T, SW1353, ATDC5, PLAT-A
Authentication	The cell lines were not authenticated.
Mycoplasma contamination	All cell line were tested Mycoplasma negative.
Commonly misidentified lines (See ICLAC register)	No commonly misidentified cell lines were used in this study.

Animals and other organisms

Policy information about studies involving animals; ARRIVE guidelines recommended for reporting animal research

Laboratory animals	C57BL/6J, Wwp2 KO (Wwp2 1ins/1ins) mice, miR-140 KO (miR-140 14del/14del), miR-140/Wwp2-double KO (DKO) (Wwp2 1del/1del; miR-140 132del/132del), miR-140 -/- and Wwp2-C838A were used. All animal studies were performed according to protocols approved by the Scripps Institutional Animal Care and Use Committee. All mice were freely allowed to access to food, water and activity.
Wild animals	We did not use wild animals in this study.
Field-collected samples	We did not use filed-collected samples in this study.

Human research participants

Policy information about studies involving human research participants

Population characteristics	We used human samples, which obtained from 17-90 years old subjects. They consisted of 32 females and 26 males.
Recruitment	Human cartilage samples for histological analyses were obtained at autopsy from individual ages 19-86 years within 48 hours post-mortem under approval by the Scripps Human Subjects Committee. Human tissues were obtained with approval by the Scripps Human Subjects Committee at The Scripps Research Institute.

ChIP-seq

Data deposition

☐ Confirm that both raw and final processed data have been deposited in a public database such as GEO.

☐ Confirm that you have deposited or provided access to graph files (e.g. BED files) for the called peaks.

Data access links *May remain private before publication.*	*For "Initial submission" or "Revised version" documents, provide reviewer access links. For your "Final submission" document, provide a link to the deposited data.*
Files in database submission	*Provide a list of all files available in the database submission.*
Genome browser session (e.g. UCSC)	*Provide a link to an anonymized genome browser session for "Initial submission" and "Revised version" documents only, to enable peer review. Write "no longer applicable" for "Final submission" documents.*

Methodology

Replicates	*Describe the experimental replicates, specifying number, type and replicate agreement.*
Sequencing depth	*Describe the sequencing depth for each experiment, providing the total number of reads, uniquely mapped reads, length of reads and whether they were paired- or single-end.*
Antibodies	*Describe the antibodies used for the ChIP-seq experiments; as applicable, provide supplier name, catalog number, clone name, and lot number.*
Peak calling parameters	*Specify the command line program and parameters used for read mapping and peak calling, including the ChIP, control and index files used.*
Data quality	*Describe the methods used to ensure data quality in full detail, including how many peaks are at FDR 5% and above 5-fold enrichment.*
Software	*Describe the software used to collect and analyze the ChIP-seq data. For custom code that has been deposited into a community repository, provide accession details.*
